# Training-related changes in neural beta oscillations associated with implicit and explicit motor sequence learning

**DOI:** 10.1038/s41598-024-57285-7

**Published:** 2024-03-21

**Authors:** Susanne Dyck, Christian Klaes

**Affiliations:** 1 Department of Neurotechnology, Medical Faculty, Ruhr-University Bochum, Universitaetsstrasse 150, 44801 Bochum, Germany; 2 International Graduate School of Neuroscience, Ruhr-University Bochum, Universitaetsstrasse 150, 44801 Bochum, Germany; 3 Neurosurgery, University hospital Knappschaftskrankenhaus Bochum, In der Schornau 23-25, 44892 Bochum, Germany

**Keywords:** Neuroscience, Psychology

## Abstract

Many motor actions we perform have a sequential nature while learning a motor sequence involves both implicit and explicit processes. In this work, we developed a task design where participants concurrently learn an implicit and an explicit motor sequence across five training sessions, with EEG recordings at sessions 1 and 5. This intra-subject approach allowed us to study training-induced behavioral and neural changes specific to the explicit and implicit components. Based on previous reports of beta power modulations in sensorimotor networks related to sequence learning, we focused our analysis on beta oscillations at motor-cortical sites. On a behavioral level, substantial performance gains were evident early in learning in the explicit condition, plus slower performance gains across training sessions in both explicit and implicit sequence learning. Consistent with the behavioral trends, we observed a training-related increase in beta power in both sequence learning conditions, while the explicit condition displayed stronger beta power suppression during early learning. The initially stronger beta suppression and subsequent increase in beta power specific to the explicit component, correlated with enhanced behavioral performance, possibly reflecting higher cortical excitability. Our study suggests an involvement of motor-cortical beta oscillations in the explicit component of motor sequence learning.

## Introduction

Many actions we perform throughout the day have a sequential nature. Examples span from the morning routine of preparing a coffee to performing a dance choreography. Motor sequence learning, a form of visuomotor learning, is the process in which repeatedly used sequences of actions are optimized in terms of efficiency. Through motor practice, a series of movement elements are integrated into a single, skilled behavior^1^. Given the ubiquity of motor sequences, motor sequence learning is a tremendously important ability.

When learning a motor sequence implicit and explicit processes play a role: On the one hand, you need to acquire the individual elements of the sequence in their temporal order. This acquisition and conscious recollection of the sequence elements in their temporal order constitutes the explicit process^2,3^. On the other hand, you need to bind the elements into one skilled behavior via implicit motor sequence learning. Implicit learning is typically defined as the ability to acquire knowledge without the intention to learn, while the obtained knowledge is difficult to express^4,5^. In contrast, explicitly acquired knowledge is characterized by reportability and highly flexible usage^6^. The traditional model of skill learning proposes that motor learning initially consists of a cognitive phase, where declarative knowledge is acquired, to an automated phase^7,8^. Let’s take the example of learning to tie your shoes: Typically, the child follows the step-by-step instructions of its parent to guide the complex sequence of movements. After many repetitions of the action sequence, the movements gain fluency and become automated, not requiring actively attending to the task at hand anymore. Even though the transition from a cognitive phase to an automated phase might fit many cases of skill learning, the interplay between implicit and explicit learning processes is more complex. For example, motor learning can also occur implicitly without any awareness of the learned action sequence, which is also termed incidental learning^9,10^. A widely used example of such an implicit learning task is learning to ride a bike. Even without specific step-by-step instructions, children can learn to ride a bike by trial and error. In turn, even once they have mastered this motor skill, they are not able to verbally instruct someone else how to perform it. While implicit motor sequence learning has been reported in healthy individuals^9,11^, even severely amnesic patients can learn motor skills despite them knowing when and how they learned them^12^. Motor sequence learning is often investigated in a setting where subjects are learning the order of a sequence of actions. While the actions themselves are not difficult, the emphasis is on selecting the right action and executing it accurately, thus requiring goal selection and action selection^13^. While motor skills such as sequence learning typically improve during practice (online), motor performance also improves between practice sessions (offline) through memory consolidation^14,15^. In this process, the representation of the motor sequence is stabilized, making it less susceptible to interference^16^, while interference refers to forgetting due to the storage of similar items in memory^17^.

A widely used paradigm to study motor sequence learning in the lab environment is the Serial Reaction Time Task (SRTT), developed by Nissen and Bullemer in 1987^9^. It is a four-choice forced-response task where participants perform movements, i.e. button presses, based on spatially congruent visual cues. The subjects are instructed to react as fast as possible by pressing the button corresponding to the spatial cue. The next stimulus typically follows after a fixed, brief delay. The presented stimuli follow a repeating sequence, which is typically not disclosed to the subjects. These sequence trials are interspersed by random trials which serve as a control condition. The time between the cue presentation and the motor response, the reaction time (RT), decreases throughout the experiment. The RT reduction in the random trials is attributed to general performance improvements for example due to learning the stimulus-response mapping and can be contaminated by factors such as fatigue and motivation^13,18^. Importantly, the RT reduction is higher in the sequence condition compared to random sequences, which is attributed to sequence-specific motor learning. The sequence-specific performance improvements happen even in participants who are not aware of the presence of a sequence, let alone the element order of the sequence, which is usually probed at the end of the training session by asking subjects to recall or generate the sequence^9,11^. Even patients suffering from Korsakoff syndrome, who have severely impaired declarative memory, show a gradual decrease in RTs in the SRTT^9^, albeit their ability to learn more complex sequences of turns in a maze was deteriorated^19^. Thus, learning in the SRTT is assumed to be incidental and implicit. Notably, learning itself can not be measured in the SRTT, it is only indirectly inferred from lasting behavioral performance improvements^20^. The question of what exactly is learned in the SRTT and how the acquired knowledge is represented has been subject to many studies^21^. While initially it was suggested that an association between stimuli and responses is essential^11^, more recent work suggests that participants rather learn response-response associations^21^. Performance improvements are thought to be based on binding motor acts into unified sets of actions, in addition to more efficient action selection^22,23^.

The view of the SRTT as a purely implicit learning task has faced scrutiny^13^. A prominent issue in the field is that probing the awareness or the declarative knowledge in the SRTT is challenging, and relying on subjective reports as a valid method has been criticized^18,24^. One argument is that conscious accessibility is variable over time and it is not either there or not, but can be rather represented on a gradual scale^25^. The retention of learned sequences—especially of longer, more complex ones—can decay over time and is subject to interference from many sources such as interspersed random blocks^26^. There have been efforts to distinguish explicit and implicit contributions in the SRTT, for example by employing dual-task paradigms, where participants have to perform a secondary attention-demanding task in parallel to the SRTT^9^. While participants could learn simple sequences with distracted attention, their RTs for more complex sequences deteriorated in dual-task versus single-task conditions^10^. The notion of the SRTT as a purely implicit learning task has been further challenged in an arm-reaching version of the SRTT, where Moisello et al.^26^ showed that participants explicitly learned fragments of the implicit sequence. Besides the classical version of the SRTT^9^, explicit variants that include awareness of the presence of a sequence have been used (for example^27–30^). Directly manipulating the subjects’ awareness through instructions can circumvent the challenges associated with measuring the awareness about sequence regularities^18^.

The interaction between implicit and explicit learning is not yet fully understood. While some studies advocate a competition between implicit and explicit motor memory systems^31,32^, an increasing body of literature points to the notion that explicit and implicit learning run as independent, parallel processes^33–35^. It is noteworthy though, that explicit knowledge can emerge in implicit learning conditions. Varying experimental conditions, such as single vs. dual-task and the amount of training have been shown to influence the emergence of declarative knowledge^36–38^. Also, the structure of the learned sequence can have an influence, i.e. salient sequence parts such as simple left to right movements are more likely to be learned explicitly^39^.

Given the simplicity of the SRTT and the fast acquisition time, it is a well-suited paradigm to study motor sequence learning in the lab environment, in healthy subjects and patients, and in combination with neuroimaging. The SRTT paradigm has been widely used to study the neural basis of motor sequence learning in neuroimaging studies using PET^40^ and MRI^29,41^. However, the findings are very heterogeneous. For example, earlier studies reported striatal activation in implicit versions of the SRTT^41^ and an activation of the Dorsolateral Prefrontal Cortex (DLPFC) in explicit SRTT^42^, while Schendan et al.^29^ found learning-related activation in the striatum and DLPFC in both the explicit and implicit SRTT. Even activation of the hippocampus has been reported in both modes of learning, although the hippocampus is typically associated with explicit learning^29,43^. Most studies employ either an implicit or an explicit version of the task, while there are only a few studies (for example Schendan et al.^29^) that investigate both implicit and explicit motor sequence learning within the same subject group.

In contrast to the vast body of neuroimaging studies, there are much fewer studies focusing on the role of neural oscillations in motor sequence learning, as measured by EEG and MEG. Oscillatory activity, or synchronous activity, of a multitude of neurons at a specific rhythm has been proposed as an important mechanism for neuronal communication and cognitive processing^44,45^. The frequency of such oscillations is categorized into distinct frequency bands that are associated with specific cognitive states: delta (1–3 Hz), theta (4–7 Hz), alpha (8–12 Hz), beta (13–30 Hz) and gamma (30–100 Hz). Beta oscillations are of particular interest in motor sequence learning since they are involved in motor control and sensorimotor processing: Voluntary movements have been shown to induce changes in the beta oscillations in the sensorimotor network^46,47^, typically contralateral to the moving limb. Prior to and during movements, a decrease in beta power can be seen relative to the baseline, which is referred to as event-related desynchronization (ERD)^46^ or movement-related beta decrease^48^. 500 ms to 1 s after the movement, an increase in beta power (event-related synchronization, ERS) occurs, called the post-movement beta rebound^46^.

Beta oscillations can be altered in motor learning^49–51^, i.e. beta power modulation has been implicated in various motor skill learning tasks^49,52^ and specifically in motor sequence learning^50^. In a MEG study using an implicit SRTT, Pollok et al.^50^ reported increased beta ERD in the implicit condition compared to the random condition. Since the changes in beta ERD correlated with behavioral performance, beta ERD has been proposed as a biomarker for implicit motor sequence learning^50^. Furthermore, movement-related beta modulation has been shown to increase with prolonged practice in an arm-reaching task in healthy subjects, while this practice-related increase is reduced in patients suffering from Parkinson’s disease^52,53^. Further evidence of the role of beta oscillations in motor sequence learning stems from studies using non-invasive stimulation: Transcranial alternating current stimulation of the primary motor cortex at a frequency of 20 Hz has been shown to facilitate the learning of an implicit motor sequence and make it less susceptible to interference^54^. The change in beta power that is induced by motor learning has been associated with an increase in cortical excitability and with training-related plasticity changes in the primary motor cortex^55^. The practice-related beta power increase from early to late learning has been related to mechanisms of motor skill retention^52^. Additionally, Engel and Fries^56^ proposed that beta oscillations are stronger when the current state is intended to be maintained. In turn, beta power suppression has been proposed to represent a state of motor or cognitive readiness, related to prospective control and anticipatory mechanisms^55–57^.

Changes in beta power have been also linked to the predictability of stimuli: Movements induced by predictable rhythmic stimuli were associated with stronger beta ERD at central regions, in contrast to non-predictable random stimuli^58^. Similarly, in an EEG study where participants reacted to predictive and non-predictive preparation cues that reliably or unreliably predicted a go cue, lower beta power has been found in the contralateral sensorimotor cortex only for the case of predictive cues^59^. Furthermore, beta oscillations at the sensorimotor cortex not only play a role in the motor domain, but also in explicit learning scenarios: In an EEG study where subjects had to make semantic decisions on objects either presented in a repeating sequence or in random order, increased beta power was reported during pre-response periods for objects in the sequential condition compared to the random condition^60^.

In conclusion, beta oscillations and their modulations at the motor cortex are relevant for motor sequence learning as probed in SRTT tasks^50^. Moreover, both implicit and explicit components contribute to motor sequence learning as parallel and interacting processes^35^. Nonetheless, there is, to our knowledge, no study that contrasts beta oscillations specific to implicit and explicit processes in motor sequence learning.

Therefore, this study aims to investigate beta oscillations in implicit versus explicit motor sequence learning and how they change in early versus late training. We developed a study design where participants concurrently learn an explicit and an implicit motor sequence, allowing us an intra-subject comparison of both learning processes. We expected three different factors that contribute to learning and improved performance: general practice effects independent of sequence learning, implicit motor sequence learning, and explicit learning. While the first two occur in the implicit learning condition, all three play a role in the explicit condition. Although termed “explicit” the explicit condition of the SRTT also entails implicit learning through repeated motor practice (see Willingham et al.^61,62^). This enables us to extract behavioral improvements and changes in motor-cortical beta oscillations that are related to sequence learning (apparent in both explicit and implicit learning) and specific to the explicit component of motor sequence learning. On a behavioral level, we expected slow performance improvements reflecting implicit learning plus fast performance improvements early in learning reflecting explicit learning. This hypothesis is based on the assumption of a slow and fast component in motor learning, represented by implicit and explicit processes, respectively^13,35^. Moreover, we anticipated that these performance improvements would be complemented by parallel changes in motor-cortical beta oscillations. Based on previous reports of practice-related increases in beta modulation^52^, we expected an increase in beta power from early to late learning in both sequence learning conditions. Moreover, since beta power suppression represents a state of motor readiness and is associated with the predictability of stimuli and thus anticipation^55–57,59^, we expected a stronger beta power suppression for the explicit component. After all, participants additionally learned the sequence order in the explicit condition, facilitating the anticipation of the next sequence element and thus the next motor response.

## Methods

### Subjects

28 subjects (mean age: 22.9 ± 4.0 years, 10 male) participated in the study. 3 subjects were unable to complete the last experimental session due to illness. Consequently, the data of the remaining 25 subjects (mean age: 22.4 ± 3.7 years, 7 male) is reported. Inclusion criteria for this study were: age between 18 and 35 years, right-handedness, normal or corrected-to-normal vision, and no psychological or neurological disorders. Before participation, all subjects gave written informed consent in accordance with the Declaration of Helsinki. They were compensated for their participation either in monetary form or through course credits. This study received ethical approval from the Research Ethics Board of the Psychology Faculty at Ruhr-University Bochum, granted under approval number 405.

### Experimental design

**Figure 1:**
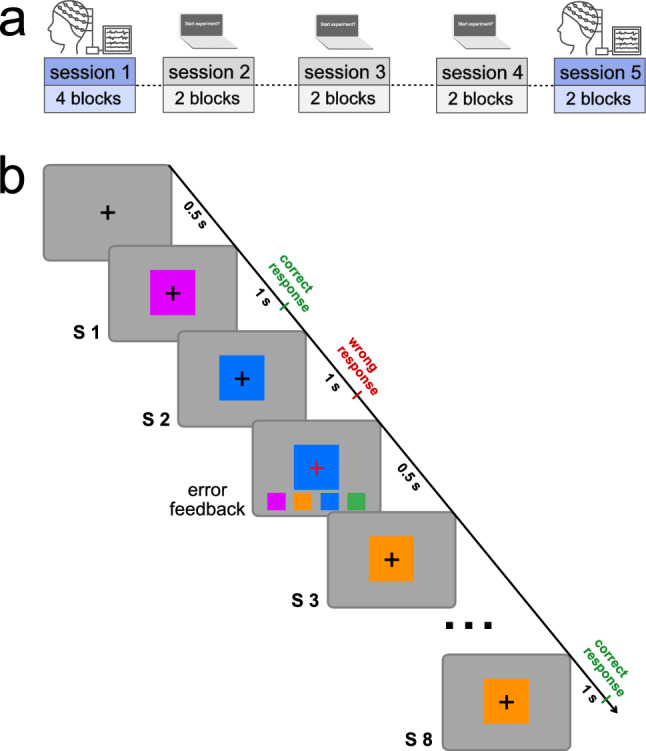
Overview of the experimental design (**a**) and the trial structure of the motor learning task (**b**). (**a**) EEG recordings have complemented the behavioral training at sessions 1 and 5, while sessions 2 to 4 took place online. (**b**) Following the presentation of a fixation cross, the first stimulus (S1) appeared and remained on screen for 1000 ms. If a correct key was pressed, the trial continued with the next stimulus (S2) which was presented for 1000 ms. In case of a wrong response, error feedback was shown for 500 ms before the trial continued with the next stimulus (S3), up until stimulus 8 (S8).

The experiment consisted of 5 training sessions, during which participants performed a motor sequence learning task. The sessions have been scheduled over the course of one week. The first and last sessions were performed in the lab, where EEG was recorded during the motor learning task. Sessions two, three, and four were online training sessions, where participants performed the same motor learning task in their home environment (for an overview, see Fig. 1a). There was one night of sleep between each training session.

#### Motor sequence learning task

The motor sequence learning task used in this experiment is a modified version of the SRTT^9^. In the original SRTT^9^, participants have to react to spatial stimuli as fast as possible by pressing a corresponding key. Moreover, some of those stimuli follow a repeating sequence, which is typically not revealed to the participant, interspersed with stimuli presentations in random order, serving as a control condition for sequence-independent learning. Instead of providing spatial cues, color stimuli have been employed to guide the motor responses in an adapted SRTT^63^. Similarly, in our task design, we used color stimuli instead of spatial cues to trigger key presses, carried out on a computer keyboard: Participants were instructed that a colored square would appear on the screen, while each color is associated with one of the 4 fingers of their right hand (thumb excluded). For simplicity, we will refer to the fingers as 1, 2, 3, 4 (1 representing the index finger, 2 representing the middle finger, etc). The color-to-key matching was shown to the participants before the experiment started: four colored squares were depicted on a horizontal line such that their spatial location indicated the respective key associated with the color stimulus. Participants were asked to press the associated finger as fast as possible once the trial started and a color stimulus appeared. The color stimulus remained on screen for a total duration of 1 s, within which the participant had to respond via a key press. If the correct key was pressed, the trial continued without any feedback by presenting the next color stimulus, again for 1 s. By contrast, a wrong or missing key press triggered error feedback for 500 ms: The fixation cross turned red and the color-to-key matching was shown as a reminder of which color stimulus is associated with which key. The trial then continued with the next color stimulus presented for 1 s. One trial consisted of 8 stimuli, with each number 1 to 4 occurring twice. Each trial started with the presentation of a fixation cross for 500 ms. Therefore, the trial duration for correctly performed sequences was 8.5 s, while the duration increased by 0.5 s per mistake made. Between trials, there was a rest period of 4 seconds.

Our task design comprised three experimental conditions: random, implicit, and explicit. In the random condition, color stimuli appeared in a pseudo-random order, serving as a control condition to assess sequence-independent learning. In both the implicit and explicit conditions, stimuli followed one of two repeating sequences, the implicit or explicit sequence, respectively. Notably, the explicit condition was distinguished by participants receiving explicit instructions tailored to this condition: Participants were informed about the presence of one repeating sequence and that the occurrence of that sequence is cued by a specific fixation-cross color (e.g. black). Importantly, the sequence itself was not reported to the participants. In trials that started with a black fixation cross, participants were asked to perform the button presses and, at the same time, remember the number of the finger that they pressed (1 for the index finger, 2 for the middle finger, etc). Those trials are the explicit motor sequence learning condition. On the other hand, when the trial started with a white fixation-cross color, participants were told that the stimuli appeared in random order and that they should simply perform the button presses as fast as possible. In reality, only 50% of those seemingly random trials were indeed stimuli in a pseudo-random order, while the other 50% followed another repeating sequence. Those hidden sequence trials that were masked as random sequences constitute the implicit condition. The task design is depicted in Fig. 1b.

The explicit sequence was 21343241 while the implicit sequence was 34132124. The sequences were designed as second-order conditional sequences, such that each element in the sequence is predicted by two preceding elements. Moreover, each element transition (for example 21) occurs in both sequences, but each triplet of elements, for example, 213 and 212, is unique to the explicit or implicit sequence, respectively. The random sequences were chosen such that: each element 1 to 4 occurred 2 times in the whole sequence; no element occurred twice in a row; no triplet of elements from the explicit or implicit sequence occurred.

The first session consisted of 4 blocks, while each block consisted of 18 sequence repetitions per condition (54 sequence trials) in a pseudo-random order. Participants were provided breaks between blocks, during which they had the opportunity to rest. The duration of each break was set to a minimum of 60 s, and participants had the autonomy to decide when to resume the experiment. The number of trials was chosen such that the training session lasted approximately 1 h. After this initial session in the EEG lab, three online sessions followed. Here, participants performed the same motor learning task in their home environment, using their home PC or laptop. They were instructed to choose a quiet spot to reduce possible disturbances as much as possible. The online sessions consisted of 2 blocks with the possibility of a break between blocks. Participants were asked to carry out the online experiment to the best of their ability and to make an effort to press the correct keys as fast as possible. They were told that their performance was tracked during the online experiment to ensure data quality. At the end of the experiment, a numerical code was depicted on the screen and participants were instructed to send this code via email to the experimenter. The code consisted of the participant ID, the session number, and the average performance. This way, we could control that participants performed the online experiment at the right time point with sufficient performance, even without supervising the experiment in the lab. The last training session, session 5, was performed in the lab again to complement this late training session with EEG recordings. Here, participants trained in the motor sequence learning task for 2 blocks. Afterwards, the stimulus-response mapping changed (2 sequence elements were exchanged) and participants performed the motor sequence learning task with the new stimulus-response mapping. While this last experimental part is not part of this report, it serves as the underlying rationale for our choice of color stimuli over spatial cues, which would not easily allow re-mapping. Moreover, it explains the reduced number of training blocks (2) in the second EEG session compared to the first one (4), since we wanted to adhere to a total training duration of 1.5 h in session 5.

The experiment was presented via PsychoPy^64^, with the online version hosted via PsychoPy’s Pavlovia (see^65^).

#### Explicit memory test

To assess the presence of conscious awareness regarding the implicit sequence, as well as to evaluate declarative knowledge in both the explicit and implicit condition, we administered an explicit memory test at the end of session 1 and session 5. The memory test consisted of a triplet recognition task, similar to the one in Schendan et al.^29^. 3 numbers were presented on screen and participants were asked to indicate how familiar those numbers are on a scale from 1 to 4. The value 1 represented the rating “definitely new”, 2 “probably new”, 3 “probably old/seen”, 4 “definitely old/seen”. Participants were asked to refrain from performing the shown sequence snippet by finger movements (either real or imaginary movements) to not contaminate the memory ratings with procedural memory. One-third of the presented numbers were triplets from the explicit sequence, one-third were from the implicit sequence. Each possible combination of triplets from the explicit and implicit sequence occurred once, resulting in 8 trials per condition. The remaining third was a random combination of sequence elements into triplets, that did neither belong to the explicit nor implicit sequence. To get a better estimate of the explicit knowledge specific to each condition, we furthermore subtracted the mean recall rating of the implicit condition and the random condition from the explicit condition, and the mean recall rating of the random condition from the implicit condition.

### EEG recordings and preprocessing

EEG has been recorded using a 64-electrode EEG system in an extended 10/20 montage (BrainAmp Standard, EasyCap). The reference and ground electrodes were fixed on the EEG cap at positions AFz and FCz, respectively. One electrode (IO) has been placed above the right eye of the participant to capture eye-movement-related artifacts. Impedances were kept below 5 k$\Omega$. The signals were recorded with a sampling rate of 500 Hz. The participants were in an electromagnetically shielded room during the EEG recordings.

The preprocessing of the obtained EEG data has been done in EEGLab^66^. After importing the raw EEG files and the channel locations, the data has been re-referenced to the average of all electrodes (IO excluded). A high pass filter of 1 Hz and a low pass filter of 40 Hz has been applied. We manually removed noisy channels and data portions that were contaminated by artifacts from the original data. To remove eye blinks and other movement-related artifacts, the data was decomposed by an independent component analysis (ICA) and components were classified as eye or muscle artifacts using EEGLab’s ICLabel algorithm^67^ plus visual inspection. Finally, we extracted epochs from the continuous data using a time interval from − 1 to 9 s with the start of the sequence trial as a synchronization event. Only correctly executed sequences have been considered. As a result, the epoch included the whole sequence of 8 button presses, with the stimulus presentation times at 500 ms, 1500 ms, 2500 ms, etc. The interval from − 1000 to − 250 ms before the start of the sequence trial has been used for baseline correction. The baseline correction was performed using a single trial normalization instead of the trial average, which has been reported to be less sensitive to noisy trials^68^.

### Data analysis and statistics

The primary measurement of learning in our modified SRTT is the reaction time. Thus, the reaction time of each key press has been measured and averaged per sequence trial to obtain the mean RT per trial. Alongside, as an additional measurement of learning, the accuracy was calculated as the number of correct button presses out of 8 button presses per trial. Moreover, the accuracy was used to filter out incorrect sequence trials (containing at least one missing or wrong key press).

Regarding the EEG data, we performed a time-frequency analysis on the motor-cortical electrodes in the left hemisphere (FC1, FC3, C1, C3, CP1, CP3), contralateral to the dominant hand of the subjects. A Morlet wavelet decomposition was applied to the sequence epochs to obtain the event-related spectral perturbation (ERSP) estimates. The ERSP gives us information about changes in the power spectra induced by an event^69^, in our case the start of the sequence trial. The ERSP estimate is obtained per subject per condition per block, with the dimensions time points of the epoch times frequencies. Once the ERSP values have been obtained from EEGLab^66^, we exported the data into Python for further processing. Subsequently, skipping the first 500 ms of each sequence trial, we applied windows of 1000 ms, spanning the duration of each visual stimulus, to average the ERSP estimates in the time domain. Thus, resulting in one ERSP value per sequence element per frequency. Since we are interested in beta oscillations, we regarded the frequencies from 13 to 30 Hz.

In the first step, we report the behavioral and neural data per condition in a group average, averaged per training session to see changes over the course of training. Moreover, since we are interested in behavioral correlates and neural oscillations specific to implicit and explicit motor sequence learning, we contrast the experimental conditions against each other. For that purpose, we follow a similar procedure as Pollok et al.^50^ used to contrast implicit learning and a control condition in an SRTT experiment; we subtract the reaction times of the implicit ($RT_\text {imp}$) and explicit ($RT_\text {exp}$) condition from the reaction times in the random condition ($RT_\text {ran}$), to obtain $\Delta RT_\text {exp}$ and $\Delta RT_\text {imp}$, respectively.

1 $$\begin{aligned} \Delta RT_\text {exp}&= RT_\text {ran} - RT_\text {exp} \nonumber \\ \Delta RT_\text {imp}&= RT_\text {ran} - RT_\text {imp}. \end{aligned}$$

Importantly, this subtraction is done block-wise and, utilizing our intra-subject design, on the level of each individual subject. Since the random condition captures sequence-unspecific learning such as learning the association between the color stimulus and the respective key press, this contrast allows us to extract the sequence-specific performance gains, for implicit and explicit motor sequence learning, respectively. Similarly, we can build the contrast between the explicit and implicit conditions:

2 $$\begin{aligned} \Delta RT_\text {ec}&= RT_\text {imp} - RT_\text {exp}. \end{aligned}$$

Through that contrast, we get the reaction times that can be attributed to the explicit component $\Delta RT_\text {ec}$ of explicit motor sequence learning. The underlying assumption here is that explicit motor sequence learning compromises implicit motor sequence learning through repeated execution plus an explicit component that is equivalent to the declarative knowledge about the sequence elements in their temporal order.

Following the same procedure, those contrasts can also be obtained for the ERSP data; we regard the contrast of the implicit or explicit sequence condition against the random control condition via block-wise subtraction

3 $$\begin{aligned} \Delta ERSP_\text {exp}&= ERSP_\text {exp} - ERSP_\text {ran}, \nonumber \\ \Delta ERSP_\text {imp}&= ERSP_\text {imp} - ERSP_\text {ran}, \end{aligned}$$

and we contrast both sequence learning conditions to see ERSP changes associated with the explicit component in explicit motor sequence learning.

4 $$\begin{aligned} \Delta ERSP_\text {ec}&= ERSP_\text {exp} - ERSP_\text {imp}. \end{aligned}$$

A Shapiro–Wilk test revealed the non-normality of the behavioral data (reaction times and recall ratings) as well as the ERSP data. To account for the non-normally distributed data, we used a generalized linear mixed model (GLMM)^70–72^ as an alternative to the repeated measures ANOVA, using the software JASP^73^. Linear mixed models have been reported to be robust enough to contrast repeated measurement conditions with varying trial ratios^74,75^ and GLMMs in particular are suitable even when the dependent variable is non-normally distributed^76^. When applicable, we used a gamma probability distribution as the underlying distribution with its default log link function. The test used in the GLMM analysis is the Likelihood ratio test. For further post-hoc analyses, we used the Wilcoxon signed-rank test as a non-parametric version of the paired sample t-test.

Furthermore, to assess whether the beta ERSP changes in the implicit and explicit condition have a behavioral relevance, we calculated the Pearson correlation between the $\Delta ERSP$ values and behavioral performance outcomes, i.e. the $\Delta ERSP$ values of each session half (session 1 first half, session 1 second half, session 5) have been correlated with the RTs and accuracy, for the explicit and implicit condition. Furthermore, to investigate the behavioral relevance of a change in beta ERSPs over the course of training, we have built the difference between the $\Delta ERSP$ at different time points as a measure of skill improvement, similar to the approach by Pollok et al.^50^: We subtracted the $\Delta ERSP$ of the first half of session 1 from the $\Delta ERSP$ of the second half of session 1 as a measure of online ERSP changes within the first training session. Similarly, we subtracted the $\Delta ERSP$ of the first half of session 1 from the $\Delta ERSP$ of session 5 as a measure of overall changes in ERSPs. This subtraction is done individually for each subject and each contrast (explicit-random, implicit-random–explicit-implicit), to obtain a measure of early vs. late ERSP changes, specific to explicit and implicit motor sequence learning. Extreme values were identified as outliers and excluded based on the interquartile range (IQR), using a multiplier of 1.5 times the IQR to determine outliers. We used JASP^73^ for the GLMM analysis and Python for the data preprocessing and, including the packages scipy^77^ and pingouin^78^, for statistical analyses (Shapiro–Wilk test and Wilcoxon signed-rank test). The correlation analysis also has been performed in Python using the packages scipy^77^ to calculate Pearson’s R and seaborn^79^ to plot the data including a regression line using a linear regression model fit.

## Results

### Behavioral data

**Figure 2:**
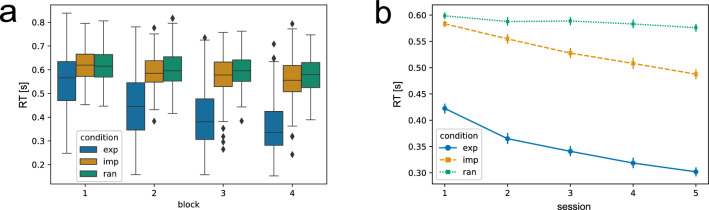
Reaction times in the explicit (exp), implicit (imp), and random (ran) conditions within the first session, across blocks (**a**), and across all training sessions (**b**) (n=25 subjects). (**a**) shows the RT per block, while one block consists of a maximum of 18 sequence trials per condition. The mean RT was averaged across the 8 key presses constituting one sequence trial and sequence trials, while only correctly executed sequences are included. The data is depicted in box plots or box-and-whisker plots. The box shows the lower and upper quartile of the data, representing 50% of the RT scores, while the whiskers extend to show the rest of the distribution. Points determined as outliers based on the interquartile range are marked as a rhombus. (**b**) Shows the mean RT across the 5 experimental sessions (i.e. average of 8 key presses). Only correct sequences are included. While the first session consists of 4 blocks, sessions 2 to 5 consist of 2 blocks, with 18 sequence trials per condition per block. The bars represent the 95% confidence intervals.

As in the original SRTT^9^, our primary measurement of learning is the reaction times (RTs). Performance gains are reflected by a reduction in RTs. First, we focus on the first training session in detail. Figure 2a shows the RTs in session 1, separated by blocks 1 to 4 to show changes throughout this initial training session. It can be seen that the RTs in the explicit condition reduce already within this first session, while the RTs in the implicit and random condition only reduce slightly.

To assess the effect of time—in this case, “block”—and experimental condition on the RTs, we used a GLMM analysis as an alternative to the repeated measures ANOVA, with the fixed factors block and condition, the dependent variable RT and subjects as the random effects grouping factor. We found a significant main effect of the factor block (${\chi }^2$ = 30.073, $p < 0.001$) and a significant main effect for condition (${\chi }^2$ = 22.562, $p < 0.001$). Furthermore, there was a significant interaction effect of block $\times$ condition (${\chi }^2$ = 23.588, $p < 0.001$). Therefore, the RT significantly varied across blocks and conditions. The comparison of the RTs in the different conditions within each block using a Wilcoxon signed-rank test shows a significant difference between the explicit and the implicit condition already in block 1 (*p* = 0.004). The difference continues to be significant in blocks 2 to 4 ($p < 0.001$). The RT difference between the explicit and random condition is not yet significant in block 1 (*p* = 0.051) but in all other blocks ($p < 0.001$). The summary of the GLMM analysis and a table showing the Wilcoxon signed-rank test results for all comparisons can be found in the Supplementary Material in Tables S1 and S2. Furthermore, an alternative depiction of the RT data within session 1, with RTs shown per sequence trial for each condition, can be also found in the Supplementary Material (Fig. S1).

Regarding the larger timescale of training sessions, a step-wise reduction of RTs is visible for the explicit and implicit condition from sessions 1 to 5 in contrast to stable RTs in the random condition (see Fig. 2b). An alternative visualization of the RTs across training sessions, including subjects’ individual data points as background data, can be found in the Supplementary Material in Fig. S2). The GLMM analysis with the factors session and condition revealed a significant main effect of condition (${\chi }^2$ = 38.218, $p < 0.001$) and session (${\chi }^2$ = 27.082, $p< 0.001$) on the dependent variable RT. Thus indicating that the experimental condition as well as the session had a significant impact on the RT. Additionally, the interaction between the condition and session was also significant (${\chi }^2$ = 22.976, *p* = 0.003), suggesting that the effect of the condition varied across different sessions. Wilcoxon signed-rank tests suggest that there is no significant difference in the RTs between the random and implicit condition in session 1 (*p* = 0.329), while the RTs in the random and the implicit condition start to show a significant difference starting from session 2 (*p* = 0.004), continuing up to session 5 ($p < 0.001$). In contrast, there is a significant difference between the RTs in the explicit condition and the RTs in the implicit and random one already in session 1 ($p < 0.001$), persisting throughout all sessions ($p < 0.001$). The GLMM summary and the Wilcoxon signed-rank test for all comparisons can be found in the Supplementary Material (Tables S3, S4).

**Figure 3:**
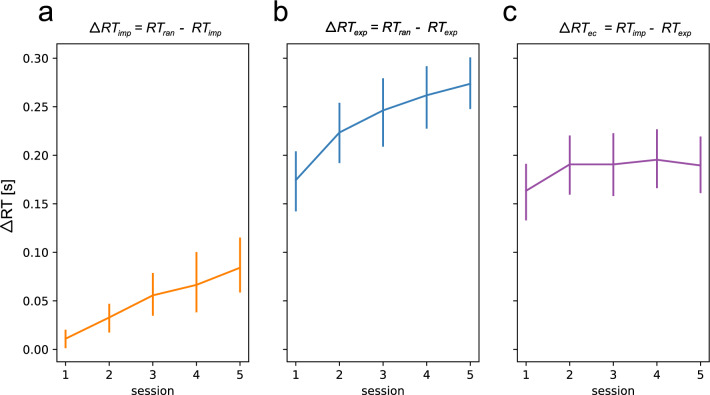
Reaction time (RT) contrasts between experimental conditions over the course of training sessions (n = 25 subjects). (**a**) Shows the difference between RTs in the random and implicit condition. (**b**) Shows the difference between RTs in the random and explicit condition. (**c**) Shows the difference between RTs in the implicit and explicit condition. The vertical bars represent the 95% confidence intervals.

In the next step, we were interested in the performance improvements (RT gains) that are specific to implicit or explicit motor sequence learning. Therefore we contrasted the RTs in the sequence learning conditions against the random control condition (see “Data analysis and statistics” in “Methods” section) to filter out sequence-independent learning effects that are captured in the random control condition. Moreover, we contrasted both sequence learning conditions against each other (see “Data analysis and statistics” in “Methods” section), based on the assumption that in the explicit condition, subjects learn explicitly plus implicitly. Therefore this contrast shows us performance gains that can be attributed to the “explicit component” or “cognitive component” of the explicit learning condition. The obtained $\Delta$ RTs averaged across training sessions are depicted in Fig. 3. The same figures including the mean $\Delta$ RT for each individual subject as background data can be found in the Supplementary Material in Fig. S3.

The sequence-learning specific RT gains in the implicit condition (Fig. 3a) are slowly developing across training sessions, starting from approx. 0 s (similar to the RTs in the random condition), reaching approx. 0.09 s on average in session 5. Similarly, the $\Delta$ RTs in the explicit condition (Fig. 3b) are increasing over the course of training sessions, from approx. 0.18 s on average in session 1 to approx. 0.27 s on average in session 5. Interestingly, the difference between the RTs in the explicit and implicit condition (Fig. 3c) is relatively constant over the course of training sessions (between 0.16 and 0.18 s on average).

**Figure 4:**
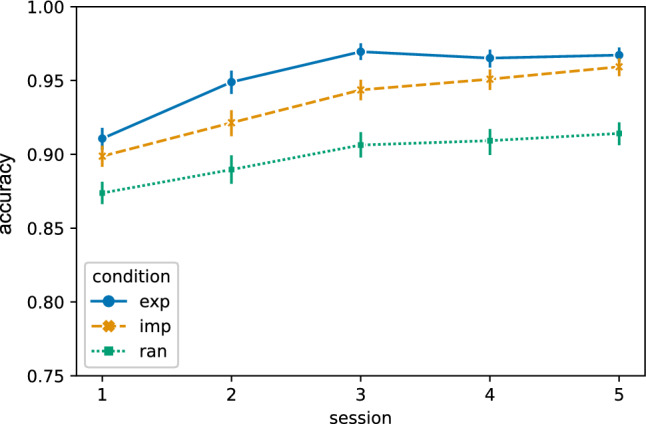
The accuracy in the explicit (exp), implicit (imp), and random (ran) condition across all 5 experimental sessions (n = 25 subjects). The accuracy in a sequence trial was calculated as the actual number of correct key presses divided by 8, which is the maximal number of correct key presses per sequence trial. Vertical lines represent the 95% confidence intervals.

A second measurement of learning, besides the RTs, is the accuracy, i.e. the number of correct key presses. Improvements in performance in our task design can be reflected in reduced RTs and in making fewer incorrect responses throughout training. Figure 4 shows that the accuracy increases in the explicit, implicit, and random conditions throughout the 5 training sessions. An equivalent figure including the mean accuracy of each individual subject as background data can be found in the Supplementary Material in Fig. S4.

To assess the effect of session and experimental condition on the accuracy, we performed a GLMM analysis, with the fixed factors session and condition, the dependent variable accuracy, and subjects as the random effects grouping factor. We found a significant main effect of the factor condition (${\chi }^2$ = 29.791, $p<$ 0.001) and a significant main effect for session (${\chi }^2$ = 16.608, *p* = 0.002). There was no significant interaction effect of condition $\times$ session (${\chi }^2$ = 8.634, *p* = 0.374). Comparing the accuracy in the different sessions and conditions using a Wilcoxon signed-rank test reveals a significant difference between the explicit and random condition across all 5 experimental sessions (all $p<$ 0.001) and between the implicit and random condition (all $p<$ 0.004). In training session 1, the accuracy between the explicit and implicit condition does not differ (*p* = 0.860), while there is a significant difference between the explicit and implicit condition in session 2 (*p* = 0.011) and session 3 (*p* = 0.001). In the last 2 training sessions, however, there is again no significant difference in the explicit and implicit condition (*p* = 0.304 in session 4; *p* = 1.000 in session 5). The summary of the GLMM analysis and a table showing the Wilcoxon signed-rank test results for all comparisons can be found in the Supplementary Material Tables S5 and S6.

### EEG data

**Figure 5:**
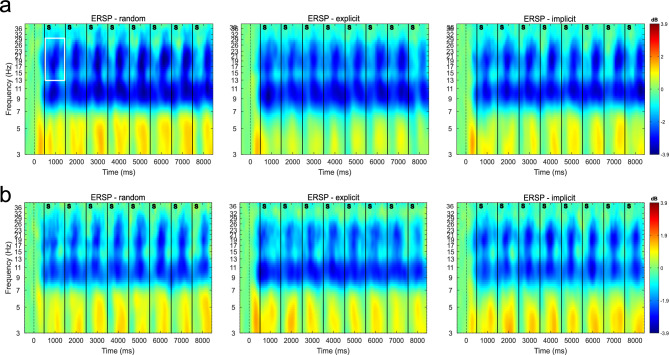
Time frequency plot showing the event-related spectral perturbation (ERSP) in the random, explicit, and implicit condition at motor-cortical electrodes, averaged over subjects (n = 25) and trials in session 1 (**a**) and in session 5 (**b**). Within the first 500 ms of the sequence trial, a fixation cross appeared, indicating if an explicit or a seemingly random (in reality: random or implicit) trial would follow. At 500 ms, 1500 ms, 2500 ms, etc, color stimuli appeared (vertical bars, “S”) and remained on screen for 1000 ms. During this time interval, participants executed the key press associated with the respective stimulus. Only correctly executed sequences were included. The y-axis shows the frequency, while the color code indicates the ERSP values in dB, with respect to a baseline period (− 1000 ms to − 250 ms before the start of the trial). For quantitative further analyses, we used the beta frequency range (13–30 Hz) and the averaged ERSP values per 1 s window (white box in the left panel of (**a**) as an example), representing the time windows of the stimuli.

EEG has been recorded at training sessions 1 and 5. Thus, time-frequency analysis of the event-related spectral perturbation (ERSP) changes in both sessions show us beta power modulations in an early vs. a late training stage, in the random control condition, and in the implicit and explicit motor sequence learning condition. A time-frequency plot of the mean ERSP at motor-cortical electrodes averaged across participants and trials, separated in experimental conditions, for session 1 and session 5 can be seen in Fig. 5. A beta power suppression can be seen in all experimental conditions following the stimulus presentation. To quantify and compare the ERSP changes, we extracted ERSP values in the beta frequency range (13–30 Hz), per experimental condition, per training session and block, per subject, and for the motor-cortical electrodes FC1, FC3, C1, C3, CP1, CP3, based on the procedure explained in “Data analysis and statistics” in the “Methods” section.

**Figure 6:**
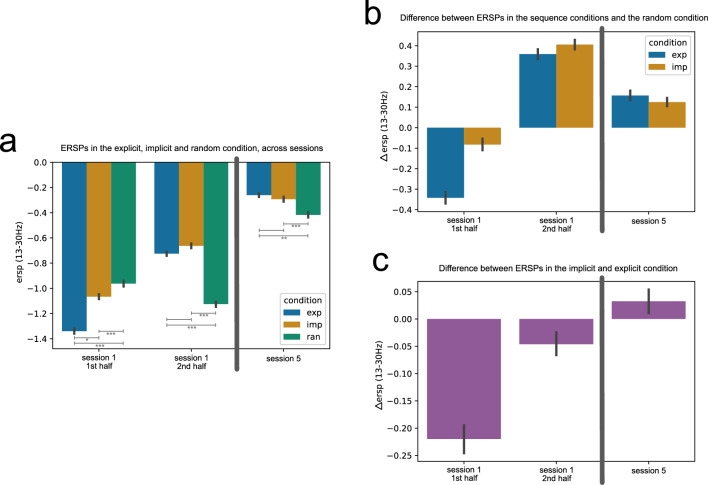
ERSPs in the explicit, implicit, and random condition (left) and differences in the ERSPs between experimental conditions (right). The ERSPs are grouped by the first and second half of session 1 and session 5 (n = 25 subjects). (**a**) The ERSPs for the explicit, implicit, and random conditions are given in dB, relative to a baseline (− 1000 to − 250 ms before the start of the sequence trial). Statistical comparisons using the Wilcoxon signed rank test are depicted with gray vertical lines, with * representing $p\le 0.05$, ** $p\le 0.01$ and *** representing $p\le 0.001$ (Bonferroni corrected for multiple comparisons). (**b**) ERSP difference between the explicit (exp, blue) and random and between the implicit (imp, orange) and random condition. The ERSP differences are given in dB, relative to a baseline (− 1000 to − 250 ms before the start of the sequence trial). The random control condition was subtracted from the sequence conditions, such that negative $\Delta$ ERSPs indicate stronger beta power suppression, while positive values indicate less beta power suppression, compared to the random condition. (**c**) ERSP difference between the explicit and implicit condition. The ERSP differences are given in dB, relative to a baseline (− 1000 to − 250 ms before the start of the sequence trial). The implicit condition was subtracted from the explicit condition, such that negative $\Delta$ ERSPs indicate stronger beta power suppression in the explicit condition, while positive values indicate stronger beta power suppression in the implicit condition. Sessions are separated by a dark-gray vertical line emphasizing different recording days. The black vertical bars represent the 95% confidence intervals. Equivalent figures including the mean ERSP and mean ERSP differences for each subject as background data can be found in the Supplementary Material in Fig. S5.

To infer ERSP changes not only in early vs. late training (session 1 vs. session 5) but also related to online learning during the initial learning phase within session 1, we separated the ERSP data of session 1 into two halves, i.e. first half (block 1 and 2) and the second half (block 3 and 4). Since session 5 consisted of only 2 blocks, those were also combined to have an equal number of blocks for all considered time points. The ERSPs in the explicit, implicit, and random condition, grouped by session 1 1st half, session 1 2nd half, and session 5 are shown in Fig. 6a.

Overall, the ERSPs are negative throughout all conditions and sessions, reflecting beta power suppression as can also be seen in the time-frequency plot, Fig. 5. Initially, the beta power suppression is higher in the explicit condition compared to the random and implicit conditions. While the ERSPs in the random condition are slightly decreasing throughout session 1, the beta ERSPs are increasing in both sequence learning conditions throughout session 1. By the end of training, in session 5, the ERSPs are less negative, reflecting a reduced beta power suppression, in all experimental conditions. We performed a GLMM analysis with the fixed factors condition (explicit, implicit, random), session half (session 1 1st half, session 1 2nd half, and session 5), and the dependent variable ERSP, using “subject” as a random grouping factor. We observed a significant main effect of the “session half” ($\upchi = 16.830, p < 0.001$), indicating that this factor had a significant impact on the ERSP measure. While the main effect of the factor condition was not significant ($\upchi = 2.033, p = 0.362$), there was a significant interaction between session half and condition ($\upchi = 11.242, p = 0.024$), suggesting that the effect of condition varied across different levels of “session half”. Further comparisons using the Wilcoxon signed-rank test show a significant difference in the ERSP values between the explicit and the implicit condition (*p* = 0.026) and between the explicit and the random condition ($p < 0.001$) in the first half of session 1. Moreover, the ERSP values between the implicit and random conditions in the first half of session 1 also show a significant difference ($p < 0.001$). However, the difference between the ERSPs in the explicit and implicit condition is not significantly different in the second half of session 1 (*p* = .906) and session 5 (*p* = 1). In contrast, there is a significant difference between the ERSP values between the explicit and random condition ($p < 0.001$) and between the implicit and random condition ($p < 0.001$) in the second half of session 1. Similarly, our test results show a significant difference in ERSPs between the explicit and random condition (*p* = 0.003) and between the implicit and random condition ($p < 0.001$) in session 5. Tables showing the summary of the GLMM analysis and the Wilcoxon signed-rank test results can be found in the Supplementary Material (Tables S7, S8).

As with the RT data, we also extract sequence-specific ERSP changes by contrasting the sequence learning conditions against the control condition, i.e. by subtracting the ERSPs of the random condition from the ERSPs in the explicit or implicit condition, respectively (see Fig. 6b).

The contrast between the sequence learning conditions and the random condition shows us a stronger beta power suppression at the early learning stage (first half of session 1) in the explicit condition. However, the beta power suppression in both sequence learning conditions is weaker in the second half of session 1, in comparison to the control condition. In session 5, the beta power suppression in the sequence learning conditions is still slightly weaker. This can be attributed to an increase in beta power in the implicit and explicit condition, as can also be seen in Fig. 6a. Moreover, it can be seen that the difference in ERSPs between both sequence learning conditions decreases after the initial learning stage. This effect can be even better observed when we regard the ERSP contrast between the explicit and implicit condition (see Fig. 6c). With this contrast, we aim at extracting the explicit or cognitive component that is part of explicit, but not implicit motor sequence learning. This contrast is only possible due to the intra-subject task design and the concurrent learning of an implicit and explicit sequence. The $\Delta$ ERSPs show that the explicit component is reflected in a stronger beta power suppression during the initial learning stage, while the difference is reduced and almost vanished in the second half of session 1 and in session 5.

To summarize the main findings: The accuracy gradually increases over the course of training sessions, while the reaction times gradually reduce, in the implicit and explicit conditions. A reduction in RT is apparent already very early in learning for the explicit condition. This early component is equivalent to the explicit or cognitive component. Reflecting the changes in behavioral performance, a similar pattern arises from the EEG data: for both implicit and explicit sequence learning, a training-induced increase in beta ERSPs can be seen. The prominent difference between both sequence learning conditions is a stronger beta power suppression in the explicit condition in the initial stage of training.

### Recall ratings

To control whether participants indeed acquired declarative or explicit knowledge about the repeating sequence in the explicit condition and not in the implicit condition, we performed a triplet recognition test at the end of training in session 1 and session 5. Here, participants rated triplets from the explicit, the implicit, and random or new sequences on a scale from 1 (definitely new) to 4 (definitely old/seen).

**Figure 7:**
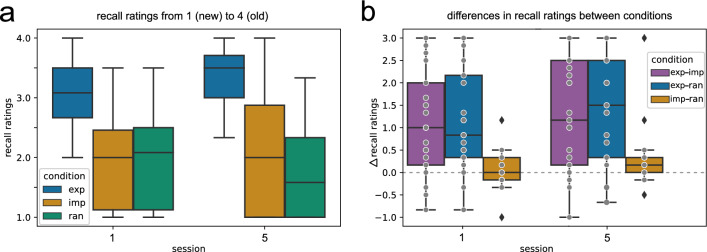
Recall ratings in the triplet recognition task for triplets in the explicit (exp), implicit (imp), and random (ran) conditions, as well as the differences in recall ratings among experimental conditions, separated in sessions 1 and 5. Ratings were ranging from 1 (definitely new) to 4 (definitely old/seen). (**a**) The mean rating per condition is shown in box plots for session 1 and session 5, respectively. The lower and upper quartile of the data is shown. (**b**) The difference in recall ratings between the explicit and implicit (exp-imp, purple), the explicit and random (exp-ran, blue), and the implicit and random (imp-ran, orange) is shown, for sessions 1 and 5. Each grey dot represents the mean rating of one subject for this particular condition and session. Outliers based on the interquartile range are marked as diamonds.

The obtained recall ratings can be seen in Fig. 7a: The mean recall rating in the explicit condition was 3.1 ± 0.6 in session 1 and 3.3 ± 0.5 in session 5. The mean recall rating in the implicit condition was 2.0 ± 0.9 in session 1 and 2.0 ± 1 in session 5, while the mean recall rating in the random condition was 1.9 ± 0.8 in session 1 and 1.7 ± 0.8 in session 5.

To compare the ratings between experimental conditions for session 1 and session 5, we used Wilcoxon signed-rank tests. In session 1, the recall ratings in the explicit condition differ significantly from the implicit condition (*p* = 0.006) and the random condition (*p* = 0.001), while the ratings between the implicit and random condition are not significantly different (*p* = 0.664). On similar terms, the recall ratings in session 5 significantly differ between the explicit and implicit condition (*p* = 0.004) and between the explicit and random condition (*p* = 0.001), while there is no significant difference between the implicit and random condition (*p* = 0.067). Moreover, the ratings between sessions 1 and 5 did not significantly differ for the explicit (*p* = 0.136), the implicit (*p* = 1.000), and the random (*p* = 1.000) condition. Table S9 in the Supplementary Material shows the Wilcoxon signed-rank test results for all comparisons. Thus, participants rated triplets from the explicit sequence overall higher compared to the implicit sequence and new triplets, while the recall ratings remained stable in the early and late training sessions. However, the high variance in the ratings, especially regarding the implicit condition in session 5, indicates a notable divergence in the levels of explicit knowledge among the participants. To focus on the individual subjects’ rating scores, we can look at the contrast between the rating scores among the experimental conditions. Figure 7b shows the differences in the rating between conditions, as obtained by subtracting the ratings of one condition, e.g. explicit, from another condition, e.g. implicit. Ideally, in accordance with the aim of our task design, the rating in the explicit condition should be greater than in the implicit and random condition. In session 1, this is the case for 19 out of 25 subjects, while 6 subjects show lower rating scores in the explicit condition compared to the implicit one. In session 5, the number of participants that show higher ratings in the explicit condition is even lower, i.e. 14 out of 25 subjects.

### Correlation analysis

**Figure 8:**
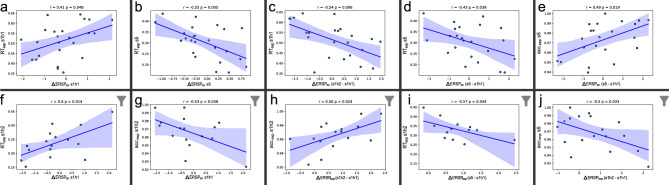
Correlation analysis between beta ERSPs and behavioral performance measures. Each dot represents the data of one particular subject. The model fit of a linear regression is shown in blue, with the 95% confidence interval as a shaded area. At the top of each panel, the Pearson r and p values are shown. $\Delta ERSP_{ec}$ represents the $ERSP_{exp}- ERSP_{imp}$, $\Delta ERSP_{exp}$ represents the $ERSP_{exp}- ERSP_{ran}$ and $\Delta ERSP_{imp}$ represents the $ERSP_{imp}- ERSP_{ran}$. The postscript s1h1 represents session 1 first half, s1h2 session 1 second half, and s5 represents session 5. (**a–e**) in the first row show the correlation analyses between $\Delta ERSP$ and RTs and accuracy (acc) including all subjects (n = 25). (**f–j**) in the second row show the correlation analyses between $\Delta ERSP$ and RTs and accuracy only including subjects, that have greater recall rating scores in the explicit condition compared to the implicit one, at the end of session 1 (n = 19); those are marked by a grey filter symbol in the top right corner. Outliers have been excluded based on the inter-quartile range, using a multiplier of 1.5.

To assess the behavioral implications of beta power modulation in the motor sequence learning task, we conducted Pearson’s correlation between the beta ERSPs and behavioral outcomes—specifically reaction times (RTs) and accuracy. We utilized the contrasted ERSP values (explicit-random, explicit–implicit, implicit-random) and correlated them with the RTs and accuracy for each condition during session 1’s first half, second half, and session 5. Additionally, we explored correlations between behavioral performance measures and training-related changes in beta ERSPs, representing changes within the first training session (the difference between $\Delta ERSP$ in session 1 first half and $\Delta ERSP$ in session 1 second half) and overall changes (the difference between $\Delta ERSP$ in session 1 first half and $\Delta ERSP$ in session 5) (see “Data analysis and statistics” in “Methods” section). Only correlations with statistical significance ($p < 0.05$) are reported. For the explicit condition, the $\Delta ERSP_{ec}$ (explicit–implicit) of the first half of session 1 shows a positive correlation with the RT in the explicit condition during this initial session half ($r = 0.41$, $p = 0.048$, see Fig. 8a). This implies that lower beta ERSP values in the explicit condition compared to the implicit one correlate with faster RTs in the first half of session 1. Conversely, in session 5, a negative correlation between $\Delta ERSP_{ec}$ and the RTs of session 5 can be seen ($r = - 0.55$, $p = 0.005$, see Fig. 8b). The difference in $\Delta ERSP_{ec}$ between the first and second half of session 1 negatively correlated with the RT in the explicit condition during the first half of session one ($r = - 0.54$, $p = 0.006$, see Fig. 8c). Similarly, the difference in $\Delta ERSP_{ec}$ between the first half of session 1 and the last training session is negatively correlated with the RTs in the explicit condition in session 5 ($r = - 0.43$, $p = 0.039$, see Fig. 8d) and positively correlated with the accuracy in the explicit condition in session 5 ($r = 0.49$, $p = 0.019$, see Fig. 8e). Thus, a lower $\Delta ERSP_{ec}$ in the first half of session 1 compared to the second half and compared to session 5 (resulting in positive $\Delta ERSP_{ec}$ values) is associated with faster initial RTs and faster RTs and higher accuracy rates at the end of training, respectively. For the implicit condition, there is no significant correlation between $\Delta ERSP_{imp}$ (implicit-random) and the behavioral measures. Interestingly, we observed that the difference in recall ratings between the explicit and implicit condition (explicit–implicit) is positively correlated with the accuracy in the explicit condition in session 5 ($r = 0.45$,$p = 0.023$, see Fig. S6 in the Supplementary Material). This suggests that participants rating triplets from the explicitly learned sequence higher than those from the implicitly learned sequence performed better at the end of training in terms of correct key presses in the explicit condition.

As reported in the recall ratings analysis (see previous section), some subjects had lower recall ratings in the explicit condition compared to the implicit condition at the end of training session 1. These subjects either failed to learn the explicit sequence explicitly or seemingly obtained explicit knowledge of the implicit motor sequence. Therefore, we excluded six subjects with lower recall ratings in the explicit condition compared to the random and implicit ones at the end of session 1 from further analysis. In the restricted subset of the remaining 19 subjects, the following significant correlations between $\Delta ERSP$ and behavioral performance measures were observed: For the explicit component, in addition to the previously reported correlations, the $\Delta ERSP_{ec}$ of the first half of session 1 shows a positive correlation with the RTs in the explicit condition in the second half of session 1 ($r = 0.60$, $p = 0.014$, see Fig. 8f) and, accordingly, a negative correlation with the accuracy in this session half ($r = - 0.53$, $p = 0.036$, see Fig. 8g). Moreover, the difference in $\Delta ERSP_{exp}$ between the first and second half of session 1 is positively correlated with the accuracy in the explicit condition in the second half of session 1 ($r = 0.56$, $p = 0.024$, see Fig. 8h). Lower $\Delta ERSP_{exp}$ in the first half compared to the second half of session 1 (which yields positive $\Delta ERSP_{exp}$ values) was associated with higher accuracy ratings in the next session half, showing an advantageous effect similar to the $\Delta ERSP_{ec}$ changes on the initial RTs (Fig. 8c). The overall change of $\Delta ERSP_{exp}$ between the first half of session 1 and the last session correlated negatively with RTs in the explicit condition of the second half of session 1 ($r = - 0.57$, $p = 0.044$, see Fig. 8i). Again, lower $\Delta ERSP_{exp}$ in the early phase (session 1 first half) compared to the last session was associated with faster RTs in the second half of session 1. Note that the explicit condition includes the implicit one plus the cognitive component. Finally, the difference in $\Delta ERSP_{imp}$ between the first and second half of session 1 showed a negative correlation with accuracy in the implicit condition in the final training session ($r = - 0.50$, $p = 0.033$, see Fig. 8j).

## Discussion

This study aimed to investigate how beta oscillations change by training, specifically in implicit and explicit motor sequence learning. Our unique task design, characterized by the concurrent learning of an implicit and an explicit motor sequence, allowed an intra-subject contrast of experimental conditions. In our modified version of the SRTT^9^, multiple processes affect the behavioral performance: Participants have to familiarize themselves with the task and the experimental setup, including learning the visuomotor association between the color stimuli and the button press. Furthermore, their motivation and fatigue can vary throughout a training session. Those factors are not specific to sequence learning and occur in all three experimental conditions (random, implicit, explicit). To resolve those confounding general practice effects and extract sequence-specific learning correlates, the implicit or explicit condition can be contrasted against the random control condition. The implicit and explicit conditions in our task design differ in terms of awareness of the sequence and declarative knowledge about the order of the sequence elements. In the explicit condition, subjects were instructed about the presence of a repeating sequence, thus learning here is intentional. Subjects received no instructions about the second repeating sequence in the implicit condition, thus learning here is incidental. It is crucial to note that although termed “explicit” motor sequence learning, the explicit condition also entails “implicit” motor sequence learning as in repeated motor practice^61,62^. Notably, while subjects were not informed about the implicit sequence, some became aware of its regularities during the learning process, as evidenced by the triplet recognition test results. As mentioned in the Introduction, awareness about sequence regularities can also emerge in a setting of implicit motor sequence learning. Nevertheless, by contrasting the implicit and explicit conditions, the explicit or cognitive component of explicit motor sequence learning can be extracted. In our discussion, we will first delve into the behavioral results, followed by a discussion of the EEG data. Subsequently, we will explore the outcomes of the triplet recognition task, placing them within the broader context of the ongoing implicit/explicit debate.

On a behavioral level, performance can improve in terms of faster reaction times (RTs) or in terms of less incorrect motor responses. Over the course of learning, participants demonstrated improved accuracy rates. While accuracy increased across all experimental conditions, it was higher in both the explicit and implicit conditions compared to the random condition. Additionally, the accuracy rates in the explicit and implicit conditions are comparable during the initial and final phases of learning. Previous studies have indicated that errors in one trial can lead to increased RTs in the subsequent trial^80^, indicating a potentially confounding effect of higher error rates on the measurement of RTs. Moreover, errors might induce increased levels of response inhibition as a strategy to avoid repeating the same mistakes in upcoming trials, while response inhibition has been shown to impact beta oscillatory dynamics in a GO/NO-GO paradigm^81^. To mitigate these potential confounding factors, our analysis on RTs and beta ERSPs focused exclusively on correct sequence trials, where participants executed all 8 key presses correctly.

Regarding the RTs, a reduction of RTs in the explicit condition is evident very early in learning, already within the first block of the first training session. This performance gain is reflected in the contrast of the implicit and explicit conditions. While established within the first session, it stays constant throughout the 5 training sessions. That leads us to assume that the explicit process plays a critical role in performance improvements very early on. Moreover, the RTs reduce throughout training sessions, in both the explicit and implicit conditions. We attribute those performance gains to the implicit component of motor sequence learning, which dominates learning in the implicit condition, but also plays a role in the explicit condition. This implicit component reduces the RTs on a slower timescale compared to the fast explicit component.

The point of a fast and slow component contributing to motor learning has been raised before, especially in the context of motor adaptation: in a prominent model by Smith et al.^82^, a learning curve is modeled by including two underlying components of learning, a fast and a slow process^13,82^. McDougle et al.^83^ suggested the fast component being equivalent to the explicit component and the slow component being equivalent to the distinct implicit component, respectively^13^. An according model of parallel processes is proposed in motor sequence learning^35^, which is in line with our behavioral data. Typically, the fast component is characterized as being learned fast and being forgotten fast. However, it is noteworthy, that despite repeated practice and automation of the explicit motor sequence, the performance gains attributed to the cognitive component do not reduce throughout the 5 training sessions. This might be related to our task design: We ask participants to remember the numbers of the fingers pressed, and that a memory test will follow at the end of session 1 and session 5. Since the sequence consists of only 8 elements, participants can typically learn the element order very fast, within the first few explicit sequence trials. Once explicit knowledge about the sequence is obtained, it is easy to retain it in the working memory, since the working memory capacity is otherwise relatively free in this task. Furthermore, since the stimuli are paced in 1-s intervals, participants have an incentive to react fast, therefore the explicit knowledge about the sequence order remains relevant throughout all training sessions. Moreover, the 5 training sessions in our experiment might be too less to transition to a more automated stage. Especially given that the practice trials in our experiment are split across three conditions, resulting in a reduced number of sequence repetitions per condition compared to the typically used version of the SRTT^9^.

Regarding the EEG data, we were interested in how beta power changes in early versus late training and between implicit and explicit motor sequence learning, with a focus on motor-cortical sites. For that purpose, we regarded ERSP changes at motor-cortical sites contralateral to the moving hand, at an early and late training stage (session 1 and session 5) and within the initial learning stage in session 1. A first observation that spans all experimental conditions was an increase in beta ERSPs from the early (session 1), to the late training stage (session 5). An increase in movement-related beta oscillations related to the prolonged practice of a motor task, in which participants simply performed reaching movements towards unpredictable targets, has been reported previously^53^. This might be related to a habituation effect reducing the amount of attention that is directed toward the practiced motor task^84,85^, since less attention might be needed to perform the by session 5 already well-known experiment. Furthermore, Kilavik et al.^86^ reported an increase in beta power in the pre-cue phase that is possibly related to the expectancy of the upcoming cue, while this effect was observed even when the cue had no task-related information. Since in our modified SRTT design, the stimuli appeared in fixed 1-s intervals, subjects could get used to this rhythm over time. This could have induced an expectancy of the next stimulus presentation at later training stages, in turn leading to an increase in beta power in all three experimental conditions. Such general practice effect from the early to late learning phase, irrespective of sequence learning, strains the importance of contrasting the experimental conditions against each other.

When regarding the ERSPs in the explicit and implicit condition contrasted against the random condition, an increase from the first to the second half of session 1 was apparent. This increase within session 1 might again relate to the prolonged practice of a motor task, in this case repeatedly practicing the explicit and implicit motor sequence (in contrast to the general practice of pressing the corresponding button associated with the color cue). An increased beta ERSP from the first to the second half of session 1 in the explicit condition was associated with higher accuracy rates in the second half of session 1, at least in a subset of the participants, namely those who showed higher recall ratings for triplets from the explicit condition compared to the implicit one. For the same subset of participants, however, an increased beta ERSP from the first to the second half of session 1 in the implicit condition was negatively correlated with the accuracy in the implicit condition during session 5. Suggesting that subjects that showed a decrease in beta ERSP specifically during the first session in the implicit condition, had better accuracy scores at the end of training. At the same time, most subjects showed an increase in beta ERSPs during this initial session. We advise caution when interpreting this short-term change in the beta ERSPs in the implicit condition: most participants have not yet learned the implicit sequence in terms of improved RTs during the initial training session, as the RTs between the random and implicit condition do not differ on a group level. Furthermore, the subjects could still have formed explicit knowledge about the implicit sequence in one of the subsequent four training sessions, affecting the accuracy in the last training session.

A related MEG study by Pollok et al.^50^ reported similar findings when studying motor-cortical beta oscillations in an implicit SRTT, while the SRTT was performed in two training sessions separated by a 10-min break: Faster RTs were associated with stronger beta power suppression during the second training session, thus, a decrease in beta power specific to implicit motor sequence learning was associated with superior learning. Interestingly, in the study by Pollok et al.^50^, approximately half of the subjects showed a decrease in beta power from the first to the second training session, while the other subjects showed an increase. Since those subjects also showed improved RTs, the authors suggested that beta power suppression over M1 is probably not the sole mechanism for motor consolidation^50^. Moreover, in a study using a real-world billiard task as a model of motor skill learning, Haar and Faisal^87^ reported that some subjects showed an increase while others showed a decrease in the post-movement beta rebound, suggesting that the distinct pattern might be related to different learning strategies that participants used for the task^48^. Overall, we also emphasize that although we see an increase of beta power in both explicit and implicit sequence learning within session 1 on a group level, there is high variability among subjects, which might reflect different strategies. While some might continue to simply respond to the color stimuli in a reaction mode, others might start to form associations between consecutive motor responses or even gain explicit knowledge about sequence regularities.

In a motor-reaching task, Nelson et al.^52^ reported a progressive increase of movement-related beta modulation in healthy controls, a phenomenon not evident in patients with Parkinson’s disease. The authors proposed that this increase might be associated with feed-forward mechanisms that improve performance and use-dependent plasticity mechanisms for skill retention^52^. Beta power modulation has been previously suggested to represent a neurophysiological marker of functional M1/S1 reorganization related to motor learning and early motor memory consolidation^49,50^. Similarly, Meissner et al.^55^ propose that beta ERD might be associated with an increase in cortical excitability and, in turn, with training-related plasticity changes. Short-term plasticity changes following a 45-min training session of motor sequence learning have been reported previously, measured by diffusion magnetic resonance imaging^88^.

Besides the increase in beta power associated with sequence learning independent of awareness, our results showed a stronger beta power suppression in the explicit condition compared to the implicit one during the early phase of learning. Correlating the contrasted beta ERSPs against the behavioral performance measures revealed that an initially stronger beta suppression specific to the cognitive component (explicit–implicit) was associated with faster RTs in the first half of session 1. Besides the stronger beta suppression early in learning, the contrast between the explicit and implicit conditions revealed an increase in beta power over the course of learning, i.e. within session 1 and from session 1 to 5. The overall increase in the beta ERSP specific to the cognitive component from the early to late training phase was associated with better performance at the end of training, in terms of faster RTs and a higher number of correct presses. In addition, the subset of subjects that reported higher recall ratings in the explicit condition compared to the implicit and random one after session 1, showed a correlation between the ERSP values of the cognitive component during the first half of session 1 and RTs and accuracy in the explicit condition of the second half of session 1. This suggests that lower values obtained from the ERSP contrast (explicit–implicit) possess predictive value for performance in subsequent blocks.

The explicit condition is characterized by acquiring knowledge about the sequence elements, which in turn allows anticipating the next stimulus and therefore the next motor response. It has been assumed that beta oscillations are stronger when the current state is intended to be maintained^56^. In turn, if change is expected, beta oscillations should decrease. Jenkinson and Brown^57^ propose that beta in the basal ganglia-cortical system is indicative of the probability that a new action has to be executed. They assume that beta oscillations are modulated via dopamine levels at cortical input sites to the basal ganglia^57^. In conclusion, beta power suppression has been proposed to represent a state of motor or cognitive readiness, related to prospective control and anticipatory mechanisms^55–57^. Another study has linked beta ERD to motor preparation and motor timing: movements induced by predictable rhythmic stimuli showed a stronger beta ERD at central regions, in contrast to a weaker beta ERD in non-predictable random stimuli^58^. In our case, we used a paced version of the SRTT, so time-wise the stimuli in each condition were equally predictable. However, only in the explicit condition the element identity and therefore the upcoming movement were known and plannable. Moreover, Teodoro et al.^59^ showed that beta ERD over sensorimotor cortex is associated with preparing predictable movements in healthy subjects. In contrast, patients with functional movement disorders showed no performance improvements in their study and impairment of beta desynchronization before movement, straining the relevance of beta ERD in explicit movement control^59^. Additionally, Ghilardi et al.^89^ hypothesized that changes in beta power, particularly the practice-related increase in beta power during movements, may signify the localized energy consumption facilitating plasticity processes through long-term potentiation (LTP) mechanisms.

Intriguingly, an EEG study where subjects made semantic decisions on objects that were presented either in random order or in a repeating sequence, revealed increased alpha and beta power during pre-response periods for objects in the sequential condition compared to the random condition^60^. The alpha and beta oscillations were found to have predictive value for the RTs in the current trial^60^. This suggests a role of beta oscillations not only in motor sequences but also in sequences within the cognitive domain. Furthermore, a recent review by Peter et al.^90^ on movement-related beta oscillations in neuropsychiatric disorders showed abnormalities in beta oscillations associated with aging, Alzheimer’s disease, and schizophrenia. Their findings align with the perspective of shared mechanisms between cognition and movement, that are regulated by beta modulation^90^. Our observations regarding beta ERSP changes specific to the cognitive component of explicit motor sequence learning and their correlation with improvements in behavioral performance can be aligned with these propositions. In line with the idea of beta modulation representing a capacity for use-dependent plasticity^52^, the stronger beta suppression specific to the cognitive component in the initial stages of learning may reflect higher cortical excitability. A speculative assumption could be that the stronger beta suppression reflects a state of readiness for acquiring new skills, since here subjects have the intention to learn. Lower beta power at the beginning of learning might potentially enhance the capacity for a subsequent increase in beta power, in turn enhancing the acquisition and retention of the learned sequence. This assumption could explain the superior performance in the explicit condition over the course of training.

Overall, our results align with the idea that explicit and implicit motor sequence learning can operate as parallel processes^35^, particularly evident in the explicit motor sequence learning condition where both explicit and implicit learning occur. However, explicit learning could also have contributed to the implicit motor sequence learning condition in our modified SRTT, as subjects became aware of the sequence regularities. To assess declarative knowledge, specifically the order of sequence elements, we employed a triplet recognition task at the end of session 1 and session 5. Given our task design, optimal participant ratings would involve assigning high numbers to triplets from the explicit condition and low numbers to triplets from the implicit and random conditions. On a group level, the mean rating for triplets in the explicit condition was indeed higher compared to the implicit and random condition. Contrasting the recall ratings between conditions revealed that 19 out of 25 subjects had greater recall ratings in the explicit compared to the implicit and random condition at the end of session 1, and 14 out of 25 subjects at the end of session 5. One possible explanation might be, that subjects that rated the triplets of the implicit sequence at least as high as the ones from the explicit sequence became aware of the implicit sequence. However, the difference between the recall ratings in the implicit and random conditions is comparably low for most subjects. Another possible explanation might be that those participants have not acquired sufficient explicit knowledge about the explicit sequence, or that the explicit knowledge has decayed over time and is thus reduced at the timepoint of the recall test. Lastly, the triplet recognition task might not have been sensitive or specific enough in probing the declarative knowledge about the sequence elements. We found no correlation between the recall ratings and the ERSP measures. However, we have seen that a stronger beta suppression in the explicit condition is of advantage for RTs in the current and upcoming blocks. One confounding factor might be that we asked subjects to rate if they think the three displayed numbers occurred during the training, without performing the displayed sequence triplet in finger movements. However, we could not control if subjects indeed were not performing the shown triplets (via real or imaginary movements) to test how familiar performing those specific triplets felt. Translating the shown triplets into real or imaginary finger movements could lead to increased recall ratings not specific to the declarative knowledge.

In general, detecting the emergence of declarative knowledge poses a great challenge in research on motor sequence learning. Typically, knowledge about the sequence regularities is probed at the end of the training session by asking subjects to recall or generate the sequence^9,11^, while also forced-choice recognition tests such as the triplet recognition task have been used previously^29^. Tests of declarative knowledge, which often equate to tests of awareness, have been criticized previously because they might be not sensitive and not specific enough to detect awareness^18,24^. Awareness is often assumed to be either there or not, while it has been argued that the level of awareness and conscious accessibility of learned knowledge is variable over time^25^ and should rather be viewed as a continuum^18^. The retention of learned sequences can decay over time or via interference from other sources such as interspersed random blocks^26^. Therefore, the declarative knowledge probed at the end of learning might not equal the declarative knowledge during training. To overcome the problem of awareness, one strategy is to instruct participants about the sequence regularity^18^, which we did in the explicit condition. Instructing participants about the regularity in an SRTT without providing the specific details of the regularity has been shown to increase explicit knowledge, measured by a sequence generation task^91^. In another SRTT study by Robertson et al.^92^ providing instructions about the presence of an underlying sequence in an explicit group was enough to modify the level of awareness in comparison to an implicit group with no instructions. Interestingly, Robertson et al.^92^ also reported that some participants in the implicit group gained explicit knowledge about the sequence, while not all subjects in the explicit group showed an optimal recall performance when measuring subjects’ awareness of the sequence. The issue of detecting declarative knowledge makes it difficult to control and extract the underlying learning processes in the implicit condition. Despite reports on behavioral improvements without declarative knowledge about the sequence^9^, findings of impaired SRTT performance of patients with amnesia^19,27^ and deteriorated SRTT performance under dual-task conditions^10^, regarding learning in the SRTT as purely implicit is unrealistic^13^. Not only the amount of training^36^, but also sleep is related to the emergence of explicit knowledge in an implicitly learned sequence^38,91,93^, which plays an important role in our multi-session experimental design. Lastly, a notable issue in multi-session designs of implicit learning is the restricted opportunities to acquire tests for probing declarative knowledge: Once participants are prompted to recall any encountered regularities during training, their attention becomes heightened toward recognizing such patterns in subsequent training sessions. Consequently, recall or recognition tests are typically deferred until the conclusion of the training period.

The challenge of effectively probing declarative knowledge in implicit motor sequence learning in a sensitive and specific manner remains an open question in the field. Over the decades, several models have been developed to explain the different learning systems involved. Avoiding the implicit/explicit debate, some models focus on the role of cognitive control processes in motor sequence learning and performance: During sequential learning, cognitive control processes can contribute towards performance improvements by regulating attention, working memory, and executive functions such as response selection^94,95^. Tubau et al.^96^ propose that the control of sequence performance can be stimulus-based, where the cognitive system is prepared to respond to stimuli, or response-based, while response-based planning relies on an internally generated action plan and depends on cognitive control to reach learning goals^95,97^. It has been reported that the increase or decrease of top-down control, achieved through a cognitive task such as focused-attention meditation, can bias participants’ performance and learning in a subsequent SRTT towards more stimulus- or response-based planning, respectively^95^. Similarly, as the model by Tubau et al.^96^, Abrahamse et al.^98^ describe a strategy shift from a reaction mode during early learning to an associative mode in later learning, which favors an increased sequence automatization. Moreover, in a prominent framework, Verwey et al.^99^ proposed that sequence execution can be controlled by a central processor and a motor processor. The central processor uses central-symbolic representations, while the motor processor uses sequence-specific motor representations. This framework also partially relates to the Dual-System Model of Keele et al.^94^ which consists of two learning systems, a multidimensional system and a unidimensional or intra-dimensional system. The former is capable of associations spanning multiple dimensions and could associate for example motor representations with higher-order representations of sequence order and thus supports the development of explicit knowledge (also see Willingham et al.^61^)^94^. On the contrary, the latter learning system could represent implicit sequence learning by associating motor responses between consecutive movements.

In conclusion, the contrast of implicit and explicit motor sequence learning realized in an intra-subject manner in our modified SRTT task, revealed, on a behavioral level, a slow component of learning in the explicit and implicit condition, representing gradual implicit sequence learning, and a fast component that is specific to the cognitive/explicit component in the explicit condition. Both components contributed to an increase in performance, especially by faster RTs. On the neural level, a stronger beta power suppression in the early phase of learning specific for the cognitive component in explicit motor sequence learning, is apparent. Additionally, throughout training, an increase in the beta ERSP could be observed which was associated with increased performance at the later training stage. On the other hand, in the implicit learning conditions, the level of beta ERSPs increased within the initial training session and decreased from early to late learning on a group level. Paralleling the behavioral data, various components can be observed that affect the motor-cortical beta ERSPs: General practice effects result in an increase in beta ERSPs from early to late learning, while the explicit component is characterized by a stronger beta suppression early on, followed by an increase in beta ERSPs. For the behavioral data, implicit and explicit learning impact the RTs in the same direction, causing a reduction in RTs. On the other hand, for the ERSP data, the effects of implicit and explicit learning mechanisms and sequence-independent effects might lead to an increase or a decrease in beta power at different time points, all contributing to the observed changes in beta ERSP. In a review focusing on beta oscillations in the sensorimotor cortex, Kilavik et al.^86^ mentioned that several components (postural maintenance, signal expectancy, signal processing, motor readiness), can contribute to producing observed changes in beta power. Thus, especially the changes in beta ERSPs that can be attributed to implicit motor sequence learning are not fully comprehensible, since here different mechanisms might play a role. On a group level, an initial increase within session 1 plus a decrease from early to late training is observable. However, in the implicit/incidental learning condition, subjects might follow different strategies to execute the sequences. The matter is even more complicated since some subjects potentially gained explicit knowledge in the implicit learning condition. This issue is ubiquitous in motor sequence learning research. Nevertheless, our task design allowed an intra-subject contrast between the explicit and implicit conditions, therefore extracting the cognitive component. Based on our data we assume that explicit learning is happening early on, reflected by a fast reduction in RTs and by stronger beta power suppression, while the explicit behavioral performance gains remain as a “jump-start” throughout learning. The stronger beta power in the explicit condition might be related to anticipation and pre-planning of the upcoming movement. Moreover, it might reflect a state of the intention to learn, inducing plasticity mechanisms to learn and retain the explicit sequence. We propose that after and during the fast explicit knowledge acquisition, associations between consecutive motor responses are strengthened via implicit learning in parallel, which is reflected in the slow RT reduction over sessions and the stepwise beta power increase in both sequence learning conditions.

Given that our task design involves the concurrent learning of both implicit and explicit motor sequences, it is essential to address potential confounding factors, particularly concerning the interleaved practice schedule. Two noteworthy considerations arise: Firstly, critics may contend that the interleaving of sequence trials across implicit, explicit, and random conditions could be subject to task-switching effects. A switch cost had been reported when subjects had to change responses based on distinct rules and, recently the switch cost has been shown to impact beta oscillations^100^. However, in our task design, participants consistently adhered to the same fundamental rule across all conditions-reacting to visual stimuli by pressing the corresponding key as fast as possible. In the explicit condition, participants received additional instruction to remember the keys pressed, which did not counteract the primary rule but rather supported it by facilitating the motor response. Moreover, rather than different rules, our task design incorporates distinct response modes, shifting from a reactive mode in the random and implicit conditions to an anticipatory mode of response initiation in the explicit condition (and potentially in the later training stage of the implicit condition with the emergence of declarative knowledge)^26^. Each sequence trial commenced with a preparatory period of 0.5 seconds, indicating if the explicit sequence would follow. Additionally, a 4-s break separated each sequence trial, allowing participants time to reset. A second argument in a similar vein suggests that the learning of the two sequences might interfere with each other. However, past studies have demonstrated the feasibility of learning two visuomotor sequences in immediate succession^101^ or even simultaneously^102,103^. Notably, an interleaved practice schedule between implicit and random sequences is advantageous for retention compared to a blocked practice design^104^. Moreover, Esser and Haider^6^ showed that presenting random and sequence trials in a blocked manner led to more explicit knowledge compared to randomly mixing sequential and random trials^97^, which we wanted to avoid given that we also included an implicit sequence learning condition.

One possible limitation of our experimental design might be that we used a paced version of the SRTT^9^. Typically, in the commonly used version of the SRTT, a motor response triggers the presentation of the next spatial cue immediately or following a fixed, short delay. In our study design, the time interval between stimuli remained fixed, requiring participants to react within the 1 s window between stimulus presentations. A fixed time interval between stimulus presentations has been employed previously by Albouy et al.^15^ in an SRTT design relying on oculomotor movements rather than key presses. Consequently, the response-to-stimulus interval (RSI) varies in each trial, and previous research has demonstrated that the RSI impacts the amount of explicit knowledge acquired^5^. Notably, in the explicit condition, shorter reaction times (RTs) lead to a correspondingly longer RSI, promoting explicit learning^5^. On the other hand, even in the implicit condition, the RSI gradually increases throughout the learning process, potentially increasing the likelihood of subjects becoming aware of the sequence regularities. However, when examining the RTs in the implicit condition, RSIs in the implicit condition ranged from 400 ms in session 1 to 500 ms in session 5, at the group level, differing only by 100 ms from the random condition by the end of training. The advantage of using a paced version is a fixed trial length which simplifies the time-frequency analysis of the EEG data. Furthermore, by limiting the possible reaction time to maximally 1 s, and punishing responses that exceeded that maximum duration, participants were challenged and had an incentive to maintain high levels of attention throughout the training sessions. Another aspect is, that we aimed to keep the visual stream constant across all training stages. Although we use our modified version of the SRTT^9^ as a paradigm for motor sequence learning, as it is widely done in this field of research, also perceptual learning plays a role^18,105^. Whilst implicit perceptual learning can also contribute towards performance improvements in our task, we aimed at reducing its influence by using color stimuli that were all presented at the center of the screen, in contrast to spatially varying stimuli. Moreover, as we instructed participants to remember the specific finger they pressed, the primary emphasis was put on the motor responses rather than the retention of color stimuli sequences (no instruction was provided to remember the series of colors displayed on the screen). In agreement with Pedraza et al.^105^, we recognize that the term ’visuomotor sequence learning’ more accurately characterizes the SRTT^9^, as it encompasses both perceptual and motor components. Nevertheless, to align with prior literature and for historical context, we use the term ’motor sequence learning’, especially given our specific focus on motor-cortical beta oscillations in this study.

Furthermore, we want to emphasize that we did not distinguish a planning and execution phase of movements in this work, since we used the whole sequence trial as an epoch so that a period before the start of the sequence can be applied as a baseline. We then averaged the ERSPs across 1 s windows, representing the duration of each stimulus presentation. Within this time window, planning and execution are taking place, since we only included correctly executed sequences in our analysis. With this approach, we wanted to avoid the problem that the movements happen at different time points after the stimulus onset. Participants performed their motor response on a common computer keyboard, making it not possible to discriminate between movement initiation or movement onset and execution, which would be favorable in studying explicit and implicit components of motor sequence learning, as it is done for example in the 2D version of the SRTT described by Moisello et al.^26^.

Lastly, EEG only probes signals at the surface of the scalp. Deeper brain structures that are reported to be crucial in motor sequence learning, such as the basal ganglia^106^, can’t be targeted with EEG or MEG^107^. To make assumptions about subcortical brain structures, EEG studies should be complemented with MRI studies or findings from intracranial recordings.

In conclusion, the intra-subject task design allows the extraction of neural and behavioral correlates associated with implicit and explicit components of motor sequence learning. Our study revealed stronger beta suppression early in learning in the explicit condition compared to the implicit and random one, and a subsequent increase in beta power over the course of practice. Thus, possibly expanding the role of sensorimotor beta modulation from the motor learning domain to the cognitive component entailed in explicit motor sequence learning. To date, the distinction between implicit and explicit learning processes remains a prominent issue in the field of motor sequence learning. In implicit task designs where participants are not instructed about the presence of a sequence, explicit knowledge about the sequence can emerge. Typically, explicit sequence knowledge is only probed at the end of training with a recall or recognition test. In the case of evidence for explicit knowledge, it can not be identified when this cognitive component emerged during learning. By taking into account the strong beta power suppression that was specifically evident in the explicit condition during the early learning phase, the explicit component could be possibly identified on a trial-by-trial basis. Combined with sudden drops in reaction times, the time point of emergence of explicit knowledge in an implicit learning task could potentially be identified in future applications.

### Supplementary Information

Supplementary Information.

## Data Availability

All data generated and analyzed during the current study are available from the corresponding author upon reasonable request.
