# Supplementary material for: Training-related changes in neural beta oscillations associated with implicit and explicit motor sequence learning
Source: Sci Rep. 2024 Mar 21;14:6781. doi: 10.1038/s41598-024-57285-7 (PMC10958048; doi:10.1038/s41598-024-57285-7)
Supplement: Supplementary file 1 — Supplementary Information. [file 41598_2024_57285_MOESM1_ESM.pdf]

Training-related changes in neural beta oscillations associated with implicit and explicit motor sequence learning

SUSANNE DYCK[1,2,*] AND CHRISTIAN KLAES[1,2,3,*]

[1] *Department of Neurotechnology, Medical Faculty, Ruhr-University Bochum, Universitaetsstrasse 150, Bochum, 44801, Germany*
[2] *International Graduate School of Neuroscience, Ruhr-University Bochum, Universitaetsstrasse 150, Bochum, 44801, Germany*
[3] *Neurosurgery, University hospital Knappschaftskrankenhaus Bochum, In der Schornau 23-25, Bochum, 44892, Germany*
[*] *susanne.dyck@rub.de, christian.klaes@rub.de*

SUPPLEMENTARY MATERIAL

1. RESULTS: BEHAVIORAL DATA

Effect	df	ChiSq	p
block	3	30.073	< .001
condition	2	22.562	< .001
block * condition	6	23.588	< .001

Note. Generalized linear mixed model with gamma family and log link function.

Note. Type III Sum of Squares

Table S1. Summary of the GLMM analysis of the fixed factors condition and block on the reaction times (RTs) of the first training session. The variable 'subject' is used as a random effects grouping factor. GLMM analysis was performed in JASP [1].

	Contrast	block	A	B	W-val	p-unc	p-corr
0	block	-	1	2	6.0	0.000	0.000
1	block	-	1	3	0.0	0.000	0.000
2	block	-	1	4	1.0	0.000	0.000
3	block	-	2	3	7.0	0.000	0.000
4	block	-	2	4	2.0	0.000	0.000
5	block	-	3	4	12.0	0.000	0.000
6	condition	-	exp	imp	0.0	0.000	0.000
7	condition	-	exp	ran	0.0	0.000	0.000
8	condition	-	imp	ran	74.0	0.157	0.471
9	block * condition	1	exp	imp	20.0	0.000	0.004
10	block * condition	1	exp	ran	36.0	0.004	0.051
11	block * condition	1	imp	ran	91.0	0.412	1.000
12	block * condition	2	exp	imp	4.0	0.000	0.000
13	block * condition	2	exp	ran	7.0	0.000	0.000
14	block * condition	2	imp	ran	111.0	0.892	1.000
15	block * condition	3	exp	imp	0.0	0.000	0.000
16	block * condition	3	exp	ran	0.0	0.000	0.000
17	block * condition	3	imp	ran	57.0	0.042	0.505
18	block * condition	4	exp	imp	0.0	0.000	0.000
19	block * condition	4	exp	ran	0.0	0.000	0.000
20	block * condition	4	imp	ran	48.0	0.018	0.211

Table S2. Wilcoxon signed-rank test results for RTs in session 1, across blocks 1 to 4 and conditions.
Rows 0-5: comparison of different blocks, specified in A and B, averaged across conditions. Rows 6-8: comparison of conditions averaged across blocks. The remaining rows show the interaction between blocks and condition for all combinations of conditions in each block. The Wilcoxon W statistic (W-val), the uncorrected p-values (p-unc) of the two-tailed test and the Bonferroni corrected p-values (p-corr) are reported.

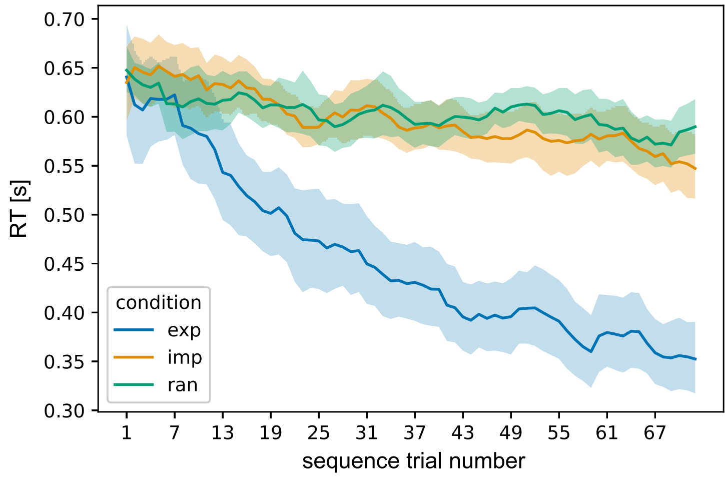

Fig. S1. Reaction times in the explicit (exp), implicit (imp) and random (ran) condition within the first session. The mean RT per sequence trial is shown, while a trial consists of 8 key presses. A sliding window approach was used to smoothen the curves (moving average smoothing, n=3). The shaded area represents the 95 % confidence interval.

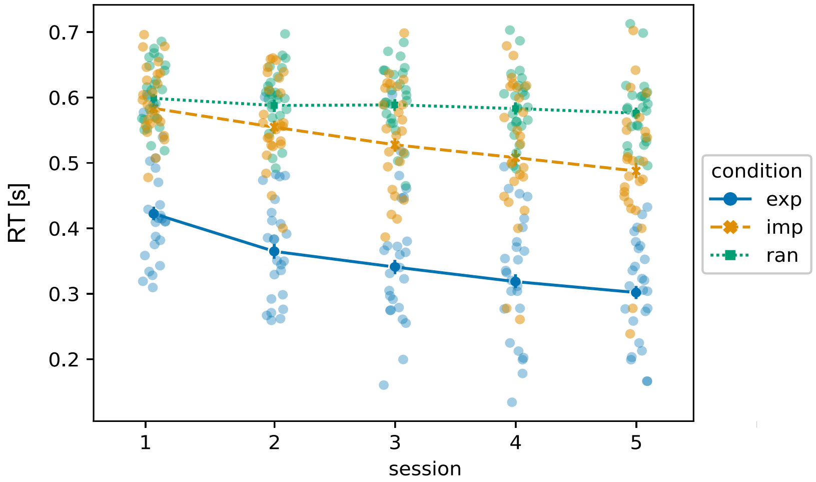

Fig. S2. Reaction times in the explicit (exp), implicit (imp) and random (ran) condition across sessions (b) (n=25 subjects). The mean RT across the 5 experimental sessions (i.e. average of 8 key presses) is shown per condition. The individual points represent the mean RT for one subject per session per condition. Only correct sequences are included. While the first session consists of 4 blocks, sessions 2 to 5 consist of 2 blocks, with 18 sequence trials per condition per block. The bars represent the 95 % confidence intervals.

Effect	df	ChiSq	p
condition	2	38.218	< .001
session	4	27.082	< .001
condition * session	8	22.976	0.003

Note. Generalized linear mixed model with gamma family and log link function.

Note. Type III Sum of Squares

Table S3. Summary of the GLMM analysis of the fixed factors condition and session on the reaction times (RTs). The variable 'subject' is used as a random effects grouping factor. GLMM analysis was performed in JASP [1].

	Contrast	session	A	B	W-val	p-unc	p-corr
0	session	-	1	2	54.0	0.003	0.025
1	session	-	1	3	47.0	0.001	0.012
2	session	-	1	4	25.0	0.000	0.001
3	session	-	1	5	1.0	0.000	0.000
4	session	-	2	3	77.0	0.020	0.203
5	session	-	2	4	63.0	0.006	0.061
6	session	-	2	5	22.0	0.000	0.000
7	session	-	3	4	73.0	0.029	0.288
8	session	-	3	5	47.0	0.001	0.012
9	session	-	4	5	17.0	0.016	0.158
10	condition	-	exp	imp	0.0	0.000	0.000
11	condition	-	exp	ran	0.0	0.000	0.000
12	condition	-	imp	ran	6.0	0.000	0.000
13	session * condition	1	exp	imp	1.0	0.000	0.000
14	session * condition	1	exp	ran	1.0	0.000	0.000
15	session * condition	1	imp	ran	78.0	0.022	0.329
16	session * condition	2	exp	imp	0.0	0.000	0.000
17	session * condition	2	exp	ran	0.0	0.000	0.000
18	session * condition	2	imp	ran	36.0	0.000	0.004
19	session * condition	3	exp	imp	0.0	0.000	0.000
20	session * condition	3	exp	ran	0.0	0.000	0.000
21	session * condition	3	imp	ran	15.0	0.000	0.000
22	session * condition	4	exp	imp	0.0	0.000	0.000
23	session * condition	4	exp	ran	0.0	0.000	0.000
24	session * condition	4	imp	ran	18.0	0.000	0.000
25	session * condition	5	exp	imp	0.0	0.000	0.000
26	session * condition	5	exp	ran	0.0	0.000	0.000
27	session * condition	5	imp	ran	1.0	0.000	0.000

Table S4. Wilcoxon signed-rank test results for RTs across sessions and conditions. Rows 0-9: comparison of different sessions, specified in A and B, averaged across conditions. Rows 10-12: comparison of conditions averaged across sessions. The remaining rows show the interaction between session and condition for all combinations of conditions in each session. The Wilcoxon W statistic (W-val), the uncorrected p-values (p-unc) of the two-tailed test and the Bonferroni corrected p-values (p-corr) are reported.

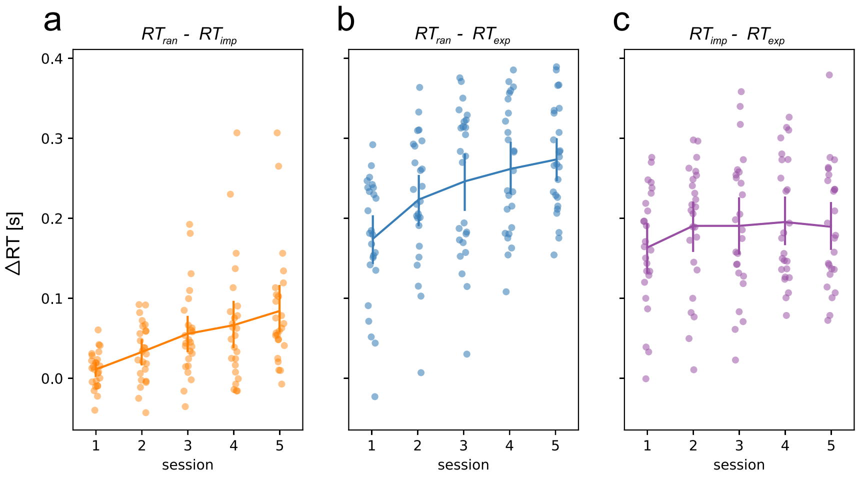

Fig. S3. Reaction time (RT) contrasts between experimental conditions over the course of training sessions (n=25 subjects). a) Shows the difference between RTs in the random and implicit condition. b) shows the difference between RTs in the random and explicit condition. c) shows the difference between RTs in the implicit and explicit condition.
The vertical bars represent the 95 % confidence intervals.
Each point in the background data represents the mean RT for one individual subject in this specific contrast and session.

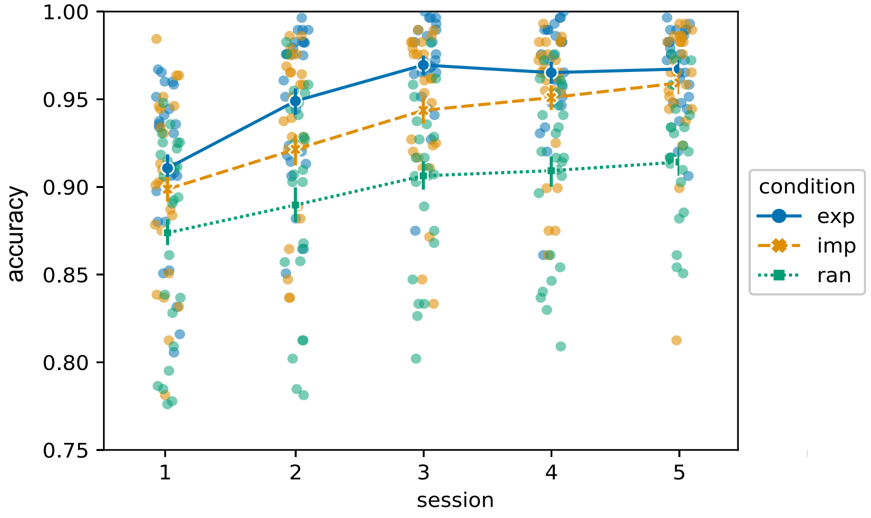

Fig. S4. The accuracy in the explicit (exp), implicit (imp) and random (ran) condition across all 5 experimental sessions (n=25 subjects). The performance in a sequence trial was calculated as the actual number of correct key presses divided by 8, which is the maximal number of correct key presses per sequence trial. The individual points represent the mean accuracy for one subject per session per condition. Vertical lines represent the 95 % confidence

Effect	df	ChiSq	p
condition	2	29.791	$< .001$
session	4	16.608	0.002
condition * session	8	8.634	0.374

Note. Generalized linear mixed model with gamma family and log link function.

Note. Type III Sum of Squares

Table S5. Summary of the GLMM analysis of the fixed factors condition and session on the performance / accuracy. The variable 'subject' is used as a random effects grouping factor. GLMM analysis was performed in JASP [1].

	Contrast	session	A	B	W-val	p-unc	p-corr
0	session	-	1	2	62.0	0.006	0.056
1	session	-	1	3	6.0	0.000	0.000
2	session	-	1	4	4.0	0.000	0.000
3	session	-	1	5	9.0	0.000	0.000
4	session	-	2	3	61.0	0.011	0.114
5	session	-	2	4	42.0	0.002	0.021
6	session	-	2	5	42.0	0.002	0.021
7	session	-	3	4	134.0	0.915	1.000
8	session	-	3	5	119.5	0.252	1.000
9	session	-	4	5	39.0	0.244	1.000
10	condition	-	exp	imp	31.5	0.000	0.000
11	condition	-	exp	ran	0.0	0.000	0.000
12	condition	-	imp	ran	0.0	0.000	0.000
13	session * condition	1	exp	imp	83.0	0.057	0.860
14	session * condition	1	exp	ran	8.5	0.000	0.000
n15	session * condition	1	imp	ran	36.0	0.000	0.004
16	session * condition	2	exp	imp	43.0	0.001	0.011
17	session * condition	2	exp	ran	0.0	0.000	0.000
18	session * condition	2	imp	ran	23.0	0.000	0.001
19	session * condition	3	exp	imp	24.0	0.000	0.001
20	session * condition	3	exp	ran	0.0	0.000	0.000
21	session * condition	3	imp	ran	3.5	0.000	0.000
22	session * condition	4	exp	imp	76.5	0.020	0.304
23	session * condition	4	exp	ran	0.0	0.000	0.000
24	session * condition	4	imp	ran	1.0	0.000	0.000
25	session * condition	5	exp	imp	93.5	0.181	1.000
26	session * condition	5	exp	ran	0.0	0.000	0.000
27	session * condition	5	imp	ran	1.0	0.000	0.000

Table S6. Wilcoxon signed-rank test results for accuracy across sessions and conditions. Rows 0-9: comparison of different sessions, specified in A and B, averaged across conditions. Rows 10-12: comparison of conditions averaged across sessions. The remaining rows show the interaction between session and condition for all combinations of conditions in each session. The Wilcoxon W statistic (W-val), the uncorrected p-values (p-unc) of the two-tailed test and the Bonferroni corrected p-values (p-corr) are reported.

2. RESULTS: EEG DATA

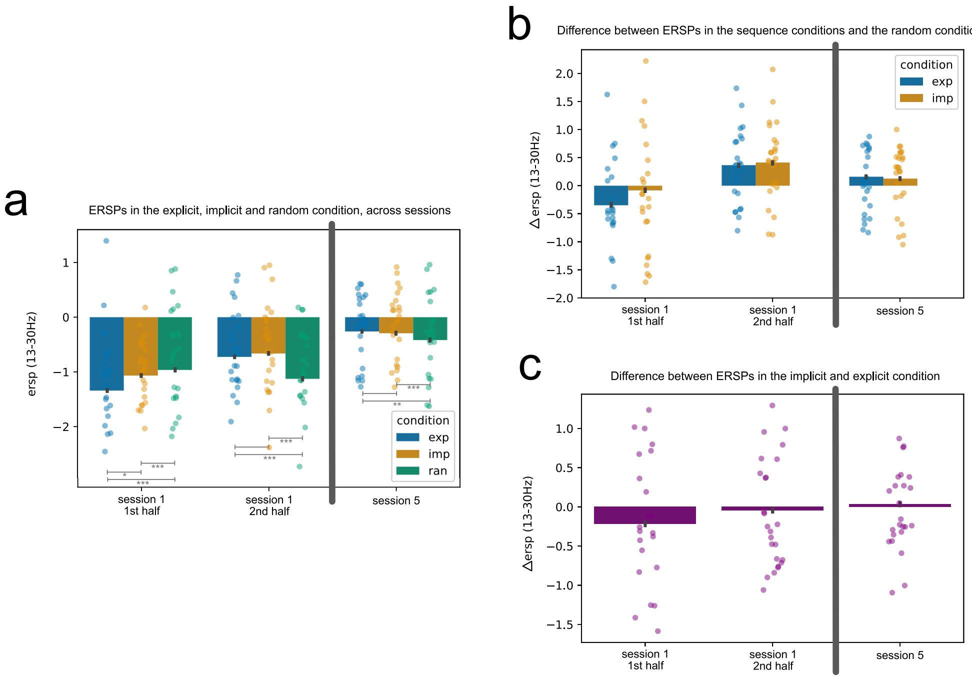

Fig. S5. Event-related spectral perturbations (ERSPs) in the explicit, implicit and random condition (left) and differences in the ERSPs between experimental conditions (right). The ERSPs are grouped by the first and second half of session 1 and session 5 (n=25 subjects). a) The ERSPs for the explicit, implicit and random condition are given in dB, relative to a baseline (-1000 to -250 ms before the start of the sequence trial). Background data points represent the mean ERSP for individual subjects, split by session half and condition. Statistical comparisons using the Wilcoxon signed rank test are depicted with gray vertical lines, with * representing $p \leq .05$, ** $p \leq .01$ and *** representing $p \leq .001$ (Bonferroni corrected for multiple comparisons). b) ERSP difference between the explicit (exp, blue) and random and between the implicit (imp, orange) and random condition. The ERSP differences are given in dB, relative to a baseline (-1000 to -250 ms before the start of the sequence trial). The random control condition was subtracted from the sequence conditions, such that negative Δ ERSPs indicate stronger beta power suppression, while positive values indicate less beta power suppression, compared to the random condition. Background data points represent the mean ERSP differences for individual subjects, split by session half and contrast (exp: explicit-random; imp: implicit-random). c) ERSP difference between the explicit and implicit condition. The ERSP differences are given in dB, relative to a baseline (-1000 to -250 ms before the start of the sequence trial). The implicit condition was subtracted from the explicit condition, such that negative Δ ERSPs indicate stronger beta power suppression in the explicit condition, while positive values indicate stronger beta power suppression in the implicit condition. Background data points represent the mean ERSP differences for individual subjects, split by session half.
Sessions are separated by a dark-gray vertical line emphasizing different recording days. The black vertical bars represent the 95 % confidence intervals.

Effect	df	ChiSq	p
condition	2	2.033	0.362
half	2	16.820	$< .001$
condition * half	4	11.242	0.024

Note. Generalized linear mixed model with gaussian family and identity link function.

Note. Type III Sum of Squares

Table S7. Summary of the GLMM analysis of the fixed factors condition and session half on the ERSP measures. The EEG data was split into halfs containing two experimental blocks: session 1 1st half, session 1 2nd half and session 5 1st half. The variable 'subject' is used as a random effects grouping factor. GLMM analysis was performed in JASP [1].

	Contrast	half	A	B	W-val	p-unc	p-corr
0	half	-	s1, 1st	s1, 2nd	311560.0	0.000	0.000
1	half	-	s1, 1st	s5, 1st	130698.0	0.000	0.000
2	half	-	s1, 2nd	s5, 1st	218003.0	0.000	0.000
3	condition	-	exp	imp	405059.0	0.000	0.001
4	condition	-	exp	ran	426142.0	0.037	0.112
5	condition	-	imp	ran	388530.0	0.000	0.000
6	half * condition	s1, 1st	exp	imp	42517.0	0.003	0.026
7	half * condition	s1, 1st	exp	ran	31842.0	0.000	0.000
8	half * condition	s1, 1st	imp	ran	34366.0	0.000	0.000
9	half * condition	s1, 2nd	exp	imp	46206.0	0.101	0.906
10	half * condition	s1, 2nd	exp	ran	28615.0	0.000	0.000
11	half * condition	s1, 2nd	imp	ran	26224.0	0.000	0.000
12	half * condition	s5, 1st	exp	imp	46665.0	0.140	1.000
13	half * condition	s5, 1st	exp	ran	40808.0	0.000	0.003
14	half * condition	s5, 1st	imp	ran	35024.0	0.000	0.000

Table S8. Wilcoxon signed-rank test results for ERSP measures across halfs (session 1 1st half, session 1 2nd half, session 5 1st half) and conditions (explicit, implicit, random). Rows 0-2: comparison of the different session halfs (s1, 1st: session 1, 1st half; s1, 2nd: session 1, 2nd half; s5, 1st: session 5, 1st half), specified in A and B, averaged across conditions. Rows 3-5: comparison of conditions averaged across session halfs. The remaining rows show the interaction between session halfs and conditions for all combinations of conditions in each session half. The Wilcoxon W statistic (W-val), the uncorrected p-values (p-unc) of the two-tailed test and the Bonferroni corrected p-values (p-corr) are reported.

3. RESULTS: RECALL RATINGS

	Contrast	session	A	B	W-val	p-unc	p-corr
0	session	-	1	5	63.5	0.125	NaN
1	condition	-	explicit	implicit	19.0	0.000	0.001
2	condition	-	explicit	random	7.0	0.000	0.000
3	condition	-	implicit	random	39.5	0.009	0.026
4	session * condition	1	explicit	implicit	29.5	0.001	0.006
5	session * condition	1	explicit	random	16.5	0.000	0.001
6	session * condition	1	implicit	random	42.5	0.111	0.664
7	session * condition	5	explicit	implicit	21.0	0.001	0.004
8	session * condition	5	explicit	random	11.5	0.000	0.000
9	session * condition	5	implicit	random	18.5	0.011	0.067
10	condition * session	explicit	1	5	51.0	0.045	0.136
11	condition * session	implicit	1	5	65.5	0.619	1.000
12	condition * session	random	1	5	65.0	0.601	1.000

Table S9. Wilcoxon signed-rank test results for the recall ratings across sessions (1,5) and conditions.
Row 0: comparison of sessions 1 and 5, specified in A and B, averaged across conditions. Rows 1-3: comparison of conditions averaged across sessions. The remaining rows show the interaction between session and condition for all combinations of conditions in session 1 and 5. Row 10-12 additionally show the contrast of session 1 and session 5 for each experimental session, obtained from a Wilcoxon signed-rank test of the recall ratings within condition and session. The column W-val reports the Wilcoxon W statistic. The uncorrected p-values (p-unc) of the two-tailed test and the Bonferroni corrected p-values (p-corr) are reported.

4. RESULTS: CORRELATION ANALYSIS

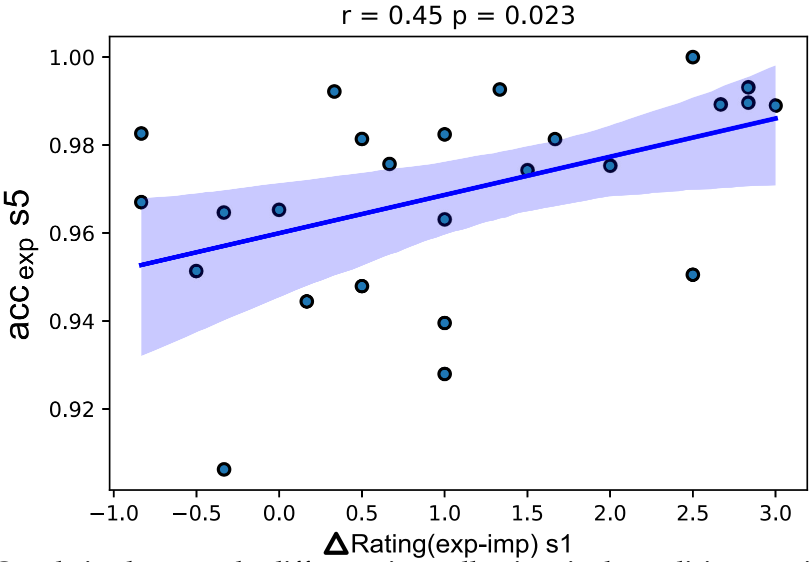

Fig. S6. Correlation between the difference in recall ratings in the explicit versus implicit condition in session 1 (ΔRating(exp-imp) s1) and the accuracy in the explicit condition in session 5 (acc_{exp}s5). Each dot represents the data of one particular subject. The model fit of a linear regression is shown in blue, with the 95 % confidence interval as shaded area. At the top of the panel the Pearson r and p value are shown.

REFERENCES

1. JASP Team, "JASP (Version 0.17.1)[Computer software]," (2023).
